# Global regulatory features of alternative splicing across tissues and within the nervous system of *C. elegans*

**DOI:** 10.1101/gr.267328.120

**Published:** 2020-12

**Authors:** Bina Koterniak, Pallavi P. Pilaka, Xicotencatl Gracida, Lisa-Marie Schneider, Iva Pritišanac, Yun Zhang, John A. Calarco

**Affiliations:** 1 Department of Cell and Systems Biology, University of Toronto, Toronto, Ontario M5S 3G5, Canada;; 2 Department of Organismal and Evolutionary Biology, Harvard University, Cambridge, Massachusetts 02138, USA;; 3 Department of Chemistry, University of Bayreuth, 95447 Bayreuth, Germany;; 4 Program in Molecular Medicine, The Hospital for Sick Children, Toronto, Ontario M5G 0A4, Canada

## Abstract

Alternative splicing plays a major role in shaping tissue-specific transcriptomes. Among the broad tissue types present in metazoans, the central nervous system contains some of the highest levels of alternative splicing. Although many documented examples of splicing differences between broad tissue types exist, there remains much to be understood about the splicing factors and the *cis* sequence elements controlling tissue and neuron subtype-specific splicing patterns. By using translating ribosome affinity purification coupled with deep-sequencing (TRAP-seq) in *Caenorhabditis elegans*, we have obtained high coverage profiles of ribosome-associated mRNA for three broad tissue classes (nervous system, muscle, and intestine) and two neuronal subtypes (dopaminergic and serotonergic neurons). We have identified hundreds of splice junctions that exhibit distinct splicing patterns between tissue types or within the nervous system. Alternative splicing events differentially regulated between tissues are more often frame-preserving, are more highly conserved across *Caenorhabditis* species, and are enriched in specific *cis* regulatory motifs, when compared with other types of exons. By using this information, we have identified a likely mechanism of splicing repression by the RNA-binding protein UNC-75/CELF via interactions with *cis* elements that overlap a 5′ splice site. Alternatively spliced exons also overlap more frequently with intrinsically disordered peptide regions than constitutive exons. Moreover, regulated exons are often shorter than constitutive exons but are flanked by longer intron sequences. Among these tissue-regulated exons are several highly conserved microexons <27 nt in length. Collectively, our results indicate a rich layer of tissue-specific gene regulation at the level of alternative splicing in *C. elegans* that parallels the evolutionary forces and constraints observed across metazoa.

Alternative precursor mRNA (pre-mRNA) splicing is a critical layer of gene expression in multicellular animals and plays a key role in increasing the functional diversity of proteins within an organism. Indeed, a significant fraction of multiexon genes undergo alternative splicing, with higher frequencies reported across vertebrates (Pan et al. 2008; Wang et al. 2008; Barbosa-Morais et al. 2012; Merkin et al. 2012) in comparison to invertebrate model organisms such as the nematode *Caenorhabditis elegans* and the fruit fly *Drosophila melanogaster* (Gerstein et al. 2010; Brown et al. 2014). Splice isoforms are often differentially expressed across tissues and/or during development (for select examples, see Pan et al. 2008; Wang et al. 2008; Gerstein et al. 2010; Ramani et al. 2011; Brown et al. 2014). As such, it remains an important goal to identify the repertoire of splice variants that help define cell- and tissue-specific functions in multicellular animals.

The relevance of alternative splicing in establishing proteomic diversity is especially reflected in the nervous system. Given its cellular complexity, the nervous system displays elevated levels of tissue-specific splicing that are also conserved throughout vertebrate evolution (Xu et al. 2002; Yeo et al. 2004; Barbosa-Morais et al. 2012; Merkin et al. 2012). More recently, transcriptome profiling in specific neuronal subtypes has revealed an even greater degree of transcript diversity generated by alternative splicing (Wamsley et al. 2018; Wang et al. 2018; Furlanis et al. 2019; Saito et al. 2019). Alternative splicing in the nervous system regulates an array of physiological events and properties, such as neurogenesis, neuronal migration, synaptic plasticity, and ion channel kinetics, and contributes to the diversity needed to fine-tune the exact activity of neurons (Ule and Darnell 2006; Norris and Calarco 2012; Raj and Blencowe 2015; Vuong et al. 2016). Alternative splicing must be tightly regulated, especially in coding sequences, to give rise to functional proteins. Splice site selection and tissue/developmental specificity are established by auxiliary *trans*-acting proteins and *cis-*acting sequence elements, which are primarily RNA-binding proteins (RBPs) and their cognate motifs, respectively (Lee and Rio 2015). Collectively, these elements direct the assembly and activity of the spliceosome and are often referred to as the “splicing code” (Wang and Burge 2008).

Although significant progress has been made in understanding the combinations of features enabling the accurate prediction of splicing outcomes (Barash et al. 2010; Zhang et al. 2010; Xiong et al. 2015; Baeza-Centurion et al. 2019; Jaganathan et al. 2019), our understanding of the splicing code remains incomplete. This fact is not surprising given that alternative splicing is regulated in a combinatorial manner, with multiple *cis* elements and/or multiple RBPs cooperating or antagonizing each other to generate splicing outcomes (Fu and Ares 2014). Additionally, the inclusion of tissue, cell, and developmental contexts is required in order to accurately predict splicing outcomes, as a number of RBPs vary correspondingly in expression patterns and as the majority of alternative splicing events are tissue and developmentally regulated (Gerstberger et al. 2014).

The nematode worm *C. elegans* possesses multiple tissue types, including a well-differentiated nervous system estimated to contain at least 118 distinct neuronal subtypes, including the major neurotransmitter-releasing classes found in vertebrates (White et al. 1986; Hobert 2010). Moreover, *C. elegans* has served as an excellent model system to study the mechanisms governing alternative splicing regulation (Zahler 2012; Gracida et al. 2016; Wani and Kuroyanagi 2017). Recent investigations harnessing the tractability of this organism have focused on whole-animal or tissue-enriched transcriptome sequencing approaches to explore changes in post-transcriptional gene regulation during development and aging (Barberan-Soler and Zahler 2008; Barberan-Soler et al. 2009; Gerstein et al. 2010; Ramani et al. 2011; Blazie et al. 2015, 2017; Ragle et al. 2015; Kaletsky et al. 2018; Kotagama et al. 2019; Warner et al. 2019; Li et al. 2020; Roach et al. 2020). However, it still remains unclear how splicing in *C. elegans* is regulated at the level of tissues, in particular at the resolution of specific neuronal subtypes. A better understanding of these mechanisms will require the systematic search for features of alternative splicing events, exploration of the evolutionary dynamics of splice isoform usage, and tools for the unbiased identification of splicing regulators.

To address these questions, we performed a genome-wide analysis of alternative splicing in *C. elegans* using broad tissue-specific and neuronal subtype–specific ribosome-associated mRNAs obtained by the translating ribosome affinity purification (TRAP) approach coupled with deep-sequencing (TRAP-seq) (Gracida and Calarco 2017). By using these data, we sought to determine the extent to which TRAP enriched tissue-specific and neuron subtype–specific splice junctions are undetected in whole-animal data. Additionally, we aimed to examine these events for distinct sequence and conservation features that may delineate mechanisms of splicing regulation.

## Results

### Tissue-enriched transcript data identifies splice junctions not identified in whole-animal profiling data

**Figure 1.**
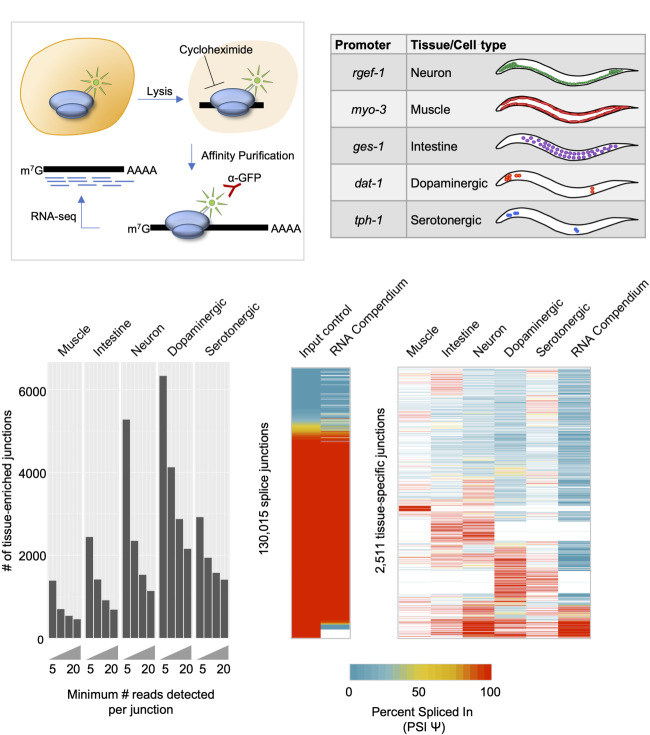
TRAP-seq identifies tissue-enriched junctions not found in whole-animal data. (*A*) Experimental outline for TRAP-seq approach. Animals expressing a GFP-tagged ribosomal protein are lysed in the presence of cycloheximide, and lysates are immunoprecipitated with anti-GFP antibodies, enriching for ribosome-associated mRNAs. cDNA libraries are prepared and then sequenced. (*B*) TRAP strains used in the present study, with reference gene listed from where promoters were cloned, as well as broad tissues or neuron subtypes for which mRNA profiles were obtained using TRAP-seq. (*C*) Number of tissue-specific splice junctions from each TRAP-seq IP sample that are absent from our whole-animal input control transcriptomes over increasing read count support thresholds. (*D*, *left*) Comparison of our whole-animal input control transcriptome data and RNA compendium data curated by Tourasse et al. (2017). PSI values for all input junctions and corresponding RNA compendium junctions are shown in the heatmap. Pearson correlation coefficient, R = 0.91, *P* < 2.2 × 10^−16^. (*Right*) PSI values of alternatively spliced tissue-specific junctions with at least 20 supporting reads, and corresponding PSI values in the RNA compendium. White indicates cases in which no reads were detected in a particular sample.

We have successfully adapted the TRAP technique (Heiman et al. 2008) for use in *C. elegans* and coupled it with deep-sequencing (TRAP-seq) (Gracida and Calarco 2017). In this technique, a green fluorescent protein (GFP) tagged ribosomal subunit protein, RPL-1, is expressed under the control of tissue- or cell-specific promoters (Fig. 1A). Whole-animal lysates are then prepared in the presence of cycloheximide, arresting ribosomes on mRNAs that are being actively translated. Ribosomes and their associated mRNAs are then immunoprecipitated from the lysate via the GFP tag, thus enriching for transcripts present in the original cell types expressing the GFP-tagged ribosomal protein. We used TRAP-seq to obtain high coverage mRNA repertoire measurements from *C. elegans* fourth-larval-stage (L4) animals for three broad tissue classes—neurons, muscle, and intestine—and two neuronal subtypes—dopaminergic and serotonergic neurons (Fig. 1B). Two biological replicates of TRAP-enriched mRNA and matched whole-animal mRNA (represented by mRNA purified from our lysates before immunoprecipitation) were collected from each transgenic strain. In total, over 2 billion uniquely mapped reads were aligned across all data sets, with summed coverage for each tissue-enriched or cell type–enriched transcriptome ranging from over 100 million to over 300 million reads (Supplemental Fig. S1).

We first mapped our TRAP-seq reads to the *C. elegans* genome and to existing gene models (for details, see Methods) and identified all aligned reads supporting annotated splice junctions, as well as high-confidence unannotated splice junctions. In total, we detected 145,811 splice junctions supported by at least five read counts across all samples (Supplemental Table S1). We next investigated whether our tissue-enriched mRNA populations enabled the detection of splice junctions that are absent owing to dilution in our whole-animal transcriptome data. In each tissue or cell type, we detected between approximately 1400 and over 6000 splice junctions supported by at least five reads that were absent from our whole-animal transcriptome data sets (Fig. 1C). Even when requiring that tissue-enriched splice junctions be supported by more than 20 reads, we still identified a total of 5577 nonredundant splice junctions not detected in our whole-animal data, spanning 1396 genes (Fig. 1C).

We next asked whether our tissue-enriched splice junctions were detected in whole-animal samples upon pooling a larger number of transcriptome data sets. To perform this analysis, we used a previously published RNA-seq compendium of reproducibly expressed splice junctions in *C. elegans*, assembled from 1682 publicly available RNA-seq experiments (Tourasse et al. 2017). Each splice junction has an associated percent spliced in (PSI) value, which is a measure of the frequency of each splice junction relative to other junctions sharing the same splice donor or acceptor sites (for details, see Methods). This schema for calculating the PSI value was applied to our data in order to compare our measurements with the RNA-seq compendium.

Both our whole-animal splice junction PSI value measurements and those of the RNA-seq compendium were strongly correlated, despite being derived from different data sets (*r* = 0.91, Pearson correlation coefficient) (Fig. 1D; left panel). However, when comparing our TRAP-seq tissue-enriched and cell type–enriched splice junctions supported by at least 20 counts with the RNA-seq compendium, we observed that ∼45% of these splice junctions are not detected in the RNA-seq compendium (Fig. 1D; right panel). We also asked whether the tissue-enriched splice junctions show distinct splicing patterns across tissues. We detected 2511 splice junctions with evidence of alternative splicing (where a minor splice variant must have a PSI value ≥5%) (Fig. 1D; right panel). Many of these splice junctions are not rare variants owing to their high PSI values and also display differentially regulated splicing patterns across tissues (Fig. 1D; right panel). Taken together, these results show that our TRAP-seq tissue-enriched mRNA profiles reveal splice junctions that are difficult to detect in whole-animal transcriptomes.

### Independent validation of TRAP-seq measurements using in vivo two-color splicing reporters

**Figure 2.**
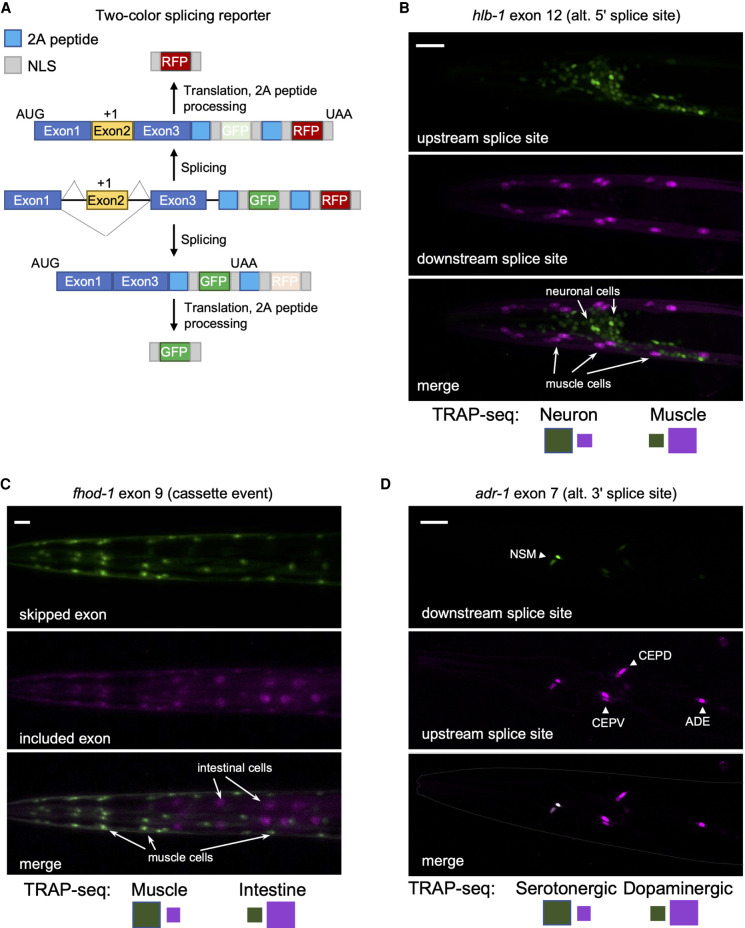
Validation of TRAP-seq measurements with two-color splicing reporters. (*A*) Schematic of the two-color splicing reporter architecture. A minigene, comprising an alternatively spliced cassette exon (yellow) with flanking introns and flanking constitutive exons (blue), is cloned upstream of GFP and mCherry (RFP) ORFs. The alternative exon is engineered to include an extra nucleotide (+1) to shift reading frame. The ORFs differ in reading frame resulting in the expression of RFP upon inclusion of the cassette exon, whereas skipping of the exon leads to the expression of GFP. Cleavage at the 2A peptides and nuclear localization sequences (NLSs) ultimately leads to free fluorescent reporter proteins to accumulate in the nucleus. (*B*) Fluorescence microscopy of splicing reporter expressed in neurons and muscle cells monitoring a splicing event in the *hlb-1* gene. *Top* labels indicate gene, alternatively spliced exon, and splicing class; *bottom* labels indicate predominant color expected in reporter for a particular tissue (larger-size green or purple square) from measured PSI values from TRAP-seq data. Scale bar, 20 μm. (*C*) Same as in *B*, with splicing reporter expressed in intestine and muscle cells monitoring splicing event in *fhod-1* gene. Scale bar, 20 μm. (*D*) Same as in *B* and *C*, with splicing reporter expressed in dopaminergic (CEP and ADE) and serotonergic (NSM) neurons monitoring splicing event in *adr-1* gene. Scale bar, 20 μm.

We next sought to validate our TRAP-seq measurements through the use of an independent approach. We used a new two-color fluorescent splicing reporter based on previous design strategies (Fig. 2A; Kuroyanagi et al. 2010; Norris et al. 2014). Specifically, these two-color reporters are designed to include an alternative exon of interest and its flanking introns and exons (referred to as a minigene). Upstream of the minigene is the promoter that drives the tissue-specific expression of the fluorescent markers. Downstream from the minigene, we inserted sequences encoding enhanced GFP (EGFP) or the red fluorescent protein mCherry, each translated in separate reading frames. The alternative exon in the minigene is then engineered to switch between both reading frames, such that when the alternative exon is skipped, EGFP is translated, and, when the exon is included, mCherry protein is produced (Fig. 2A). A similar frame-shifting strategy is applied to monitor alternative 5′ and 3′ splice site selection events (Supplemental Fig. S2A).

Additionally, our reporter included 2A peptide sequences upstream of each fluorescent protein to cleave away upstream peptides translated from the minigene exons (Ahier and Jarriault 2014). Finally, two nuclear localization signals (SV40 and EGL-13) (Lyssenko et al. 2007) were included at the N and C termini of each fluorescent protein to concentrate signal in the nucleus of each cell expressing the reporter. With this combined set of design features, these reporters enable the visualization of alternative splicing patterns by fluorescence microscopy in vivo and at single-cell resolution.

Our TRAP-seq data set was analyzed for splicing events that showed switch-like splicing patterns (a change in PSI value, or ΔPSI, of ≥80%) between the broad tissue types. Four such events were cloned into our two-color reporters (minigene sequences surrounding *hlb-1* exon 12, *fhod-1* exon 8, *zoo-1* exon 9, and *ampd-1* exon 15), and expression of these reporters was driven under the control of neuronal, body-wall muscle, or intestinal promoters (for details, see Methods) (Fig. 2B,C; Supplemental Fig. S2; see Fig. 7A). In all cases tested, we observed agreement between fluorescent reporter patterns in vivo and our TRAP-seq measurements across tissues (Fig. 2B,C; Supplemental Fig. S2). We additionally monitored the splicing patterns of exon 7 in the *adr-1* gene and exon 11 in *mlk-1*. These splicing events were identified by TRAP-seq as differentially regulated between serotonergic and dopaminergic neurons or between the neuronal subtypes and the rest of the nervous system, respectively. Again, our fluorescent reporter signals agreed with the TRAP-seq measurements (Fig. 2D; Supplemental Fig. S2). Including these reporters, a total of 14 two-color reporters were tested, and 11 reporters displayed patterns consistent with TRAP-seq measurements (Supplemental Table S2).

Taken together, although our TRAP-seq analysis and two-color reporters assess splicing patterns through different means, their general correlation strongly suggests that our TRAP-seq measurements can detect regulated splicing patterns.

### TRAP-seq identifies tissue-biased and neuronal subtype–biased splicing patterns

**Figure 3.**
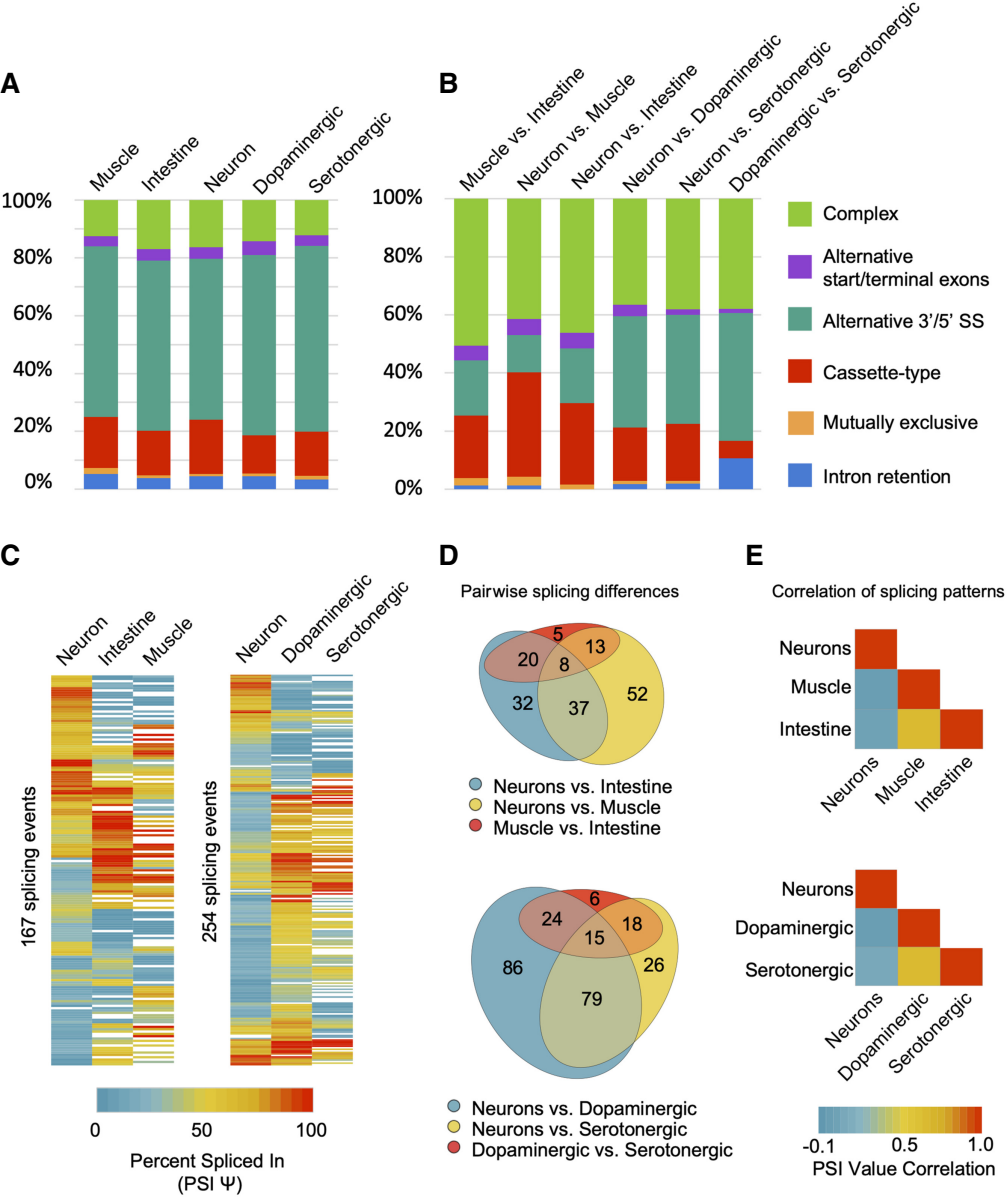
Summary and classification of tissue-regulated alternative splicing events. (*A*) Relative proportions of high-confidence alternative splicing events separated into five canonical splicing classes (listed in legend on the *right*), as well as the “complex” category comprising multiple splicing events occurring within the same gene region. Proportions in each tissue are displayed. (*B*) Relative proportions of alternative splicing events that show tissue-differential splicing outcomes between any two broad tissue or neuronal-subtype comparisons. Splicing events are grouped and colored by their class of splicing as in *A*. (*C*) Heatmaps displaying cassette, alternative 3′/5′ splice site, or alternative start/terminal exon splicing events that differ in PSI value by a minimum of 20%, between at least two of the three tissues/cell types. (*Left*) Broad tissue-specific splicing differences; (*right*) neuron-specific and neuro-subtype-specific splicing differences. (*D*) Venn diagrams representing overlap and grouping of events from heatmaps in *C*. Diagrams highlight splicing events that are differentially regulated between broad tissues (*top* diagram) or between neurons and neuronal subtypes (*bottom* diagram). (*E*) Heatmaps showing pairwise Pearson correlation values of PSI measurements for each of the broad tissue comparisons (*top* panel) and nervous system versus subtype comparisons (*bottom* panel).

We next used the MAJIQ software package (Vaquero-Garcia et al. 2016) to identify all the alternative splicing events (defined by MAJIQ as local splicing variation) in each TRAP-seq tissue transcriptome and all the tissue-differential splicing events within every pair of broad tissue types (muscle, intestine, neurons) and within the nervous system (neurons, dopaminergic neurons, serotonergic neurons; for details, see Methods) (Fig. 3). In total, we identified 2953 alternative splicing events in 2196 genes across all tissues. These alternative splicing events are distributed across five major classes: cassette type (exon skipping), alternative 3′ or 5′ splice site usage, alternative start or terminal exons, intron retention, and mutually exclusive splicing (Fig. 3A; Supplemental Fig. S3; Supplemental Table S3). We also identified splicing events that we classified as “complex” (Fig. 3A; Supplemental Fig. S3). This latter class represents splice junctions making use of more than one mode of splicing, as well as accounts for ∼10%–15% of all forms of alternative splicing in a particular tissue or cell type. As described previously for whole-animal transcriptome data (Ramani et al. 2011), the predominant class of alternative splicing in any given tissue in *C. elegans* is alternative 3′ or 5′ splice site usage (Fig. 3A).

Moreover, our analysis of tissue-biased alternative splicing identified 871 differentially regulated splicing events in 689 genes across tissues and the two neuronal subtypes (Fig. 3B; Supplemental Table S4). Again, all major classes of splicing were represented among the set of differentially regulated alternative splicing events (Fig. 3B; Supplemental Table S3). However, complex splicing events represent a much larger proportion of tissue-differential splicing compared with the distribution of possible splicing modes in a given tissue or cell type (Fig. 3, cf. A and B). For example, in the three broad tissue pairwise comparisons, complex splicing events accounted for between 40% and 50% of all differentially regulated splicing events, a significantly increased proportion compared with individual cell or tissue types (*P* values all < 1 × 10^−4^, chi-squared test) (Fig. 3, cf. A and B).

Gene Ontology (GO) overrepresentation analysis was performed on the sets of genes with exons that were differentially spliced between any two tissues. These genes with differentially spliced exons were compared against a background set of genes expressed in the same tissues (see Methods). Splicing events differentially regulated between muscle, intestine, and neurons were enriched in genes involved in actin/cytoskeleton organization, muscle cell differentiation, neuronal differentiation, and the regulation of neuronal projections (Supplemental Table S5), similar to previous findings in vertebrates (Zheng and Black 2013; Giudice et al. 2014; Raj and Blencowe 2015).

Finally, we performed clustering analysis and quantification of noncomplex, differentially regulated splicing events expressed across at least two tissues and/or cell types (Fig. 3C–E). Our analysis revealed that fewer differences in splicing were found between muscle and intestinal cells compared with differences between these two tissues and the nervous system (Fig. 3D). Similarly, fewer differences were detected between the dopaminergic and serotonergic neurons, but we identified many more differences between these neuromodulatory cells and the broad nervous system samples (Fig. 3D). Accordingly, splicing patterns were correlated between muscle and intestinal cells (*r* = 0.63) and between dopaminergic and serotonergic neurons (*r* = 0.65), but very little correlation was observed between these tissues and neuron subtypes with the broad nervous system (*r* from −0.04 to 0.07) (Fig. 3E).

Taken together, these results indicate that tissue-regulated splicing represents a substantial and complex layer of gene expression in *C. elegans*. Moreover, regulated splicing plays a particularly important role in shaping the transcriptome of the nervous system, including the fine-tuning of transcript variants expressed in dopaminergic and serotonergic neurons.

### Expression level differences and splicing pattern differences between tissues involve largely distinct sets of genes

**Figure 4.**
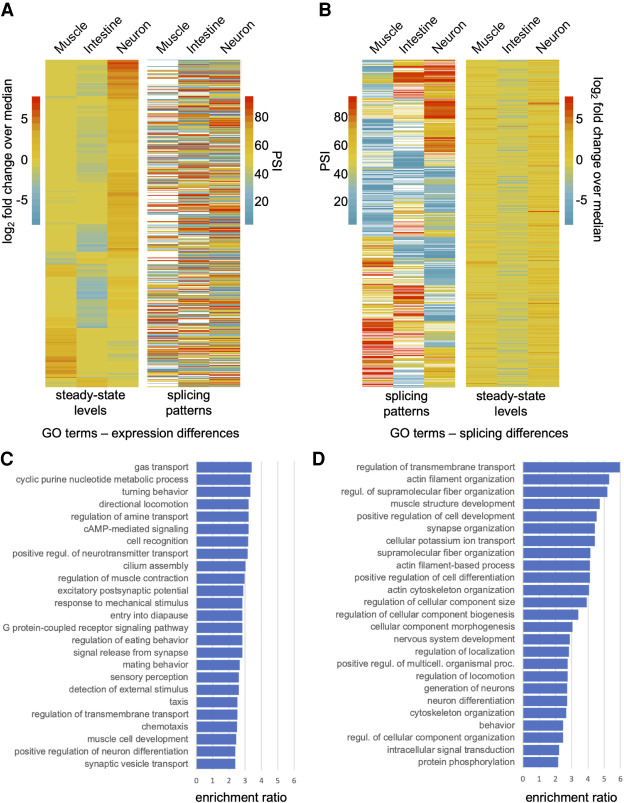
Tissue-regulated splicing and steady-state level differences are enriched in nonoverlapping gene sets. (*A*, *left*) Genes with significant steady-state transcript level differences clustered by their log_2_-transformed fold change over median ratios. (*Right*) PSI value measurements for alternative splicing events in the same genes ordered according to panel on the *left*. (*B*, *left*) Genes with tissue-regulated splicing patterns clustered by their respective PSI value measurements. (*Right*) The log_2_-transformed fold change over median ratios for the same genes with splicing differences, ordered according to panel on the *left*. (*C*) Gene Ontology (GO) enrichment analysis of genes with steady-state transcript level differences from heatmap in *A*. Overrepresentation analysis was performed applying Bonferroni correction for multiple testing. Significantly enriched biological processes with corrected *P*-values < 0.05 are shown. The enrichment ratio is the proportion of the number of genes in the sample set versus the number of genes in the background set for each GO category. Top 25 GO terms are listed. Full list is available in Supplemental Table S6. (*D*) GO enrichment analysis of genes with tissue-regulated splicing events from heatmap in *B*. Analysis is as described in *C*.

Splicing differences and steady-state mRNA differences between cell and tissue types are thought to have evolved on largely distinct sets of genes (Pan et al. 2004). To test this possibility in our tissue-specific transcriptome data, we performed a reciprocal clustering analysis of genes with differential expression or splicing patterns (Fig. 4A,B).

First, we identified all genes that were both differentially expressed between pairs of tissues and contain one or more alternative splicing events (13% of all differentially expressed genes; see Methods). After clustering genes by their differential expression patterns, we observed no obvious trends with splicing differences in the same genes (Spearman's rho values between −0.01 and 0.04, *P* > 0.3) (Fig. 4A). Similarly, genes with tissue-differential splicing events were clustered according to their PSI value (Fig. 4B). Again, there was no obvious correlation between splicing patterns and steady-state RNA level differences (Spearman's rho values between −0.04 and −0.01, *P* > 0.3) (Fig. 4B). We observed similar patterns in comparisons between the nervous system and neuronal subtypes (Supplemental Fig. S4).

Consistent with these analyses above, the top overrepresented GO categories for genes with tissue-differential splicing patterns and genes with tissue-differential expression levels were somewhat distinct (Fig. 4C,D). A wide range of biological processes are enriched in differentially expressed genes within the broad tissues, whereas differentially spliced genes are enriched in vesicle transport, actin kinetics, ion channel activity, and muscle/neuron differentiation (Fig. 4C,D; Supplemental Table S6). Taken together, our results indicate that regulated alternative splicing has contributed to tissue and cell type diversification by influencing a distinct set of genes compared with those regulated at the level of steady-state abundance.

### Tissue-regulated exons and their surrounding sequences are highly conserved across *Caenorhabditis* species

**Figure 5.**
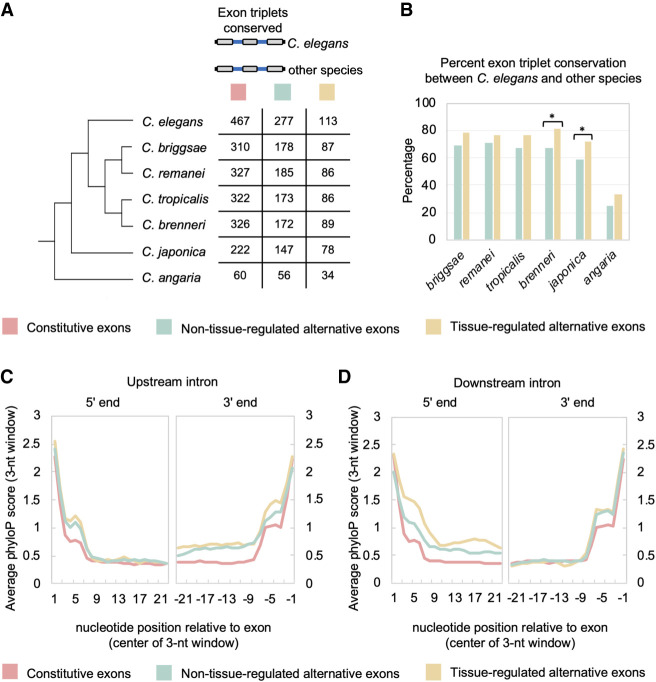
Alternative and tissue-regulated splicing are associated with increased conservation patterns. (*A*, *left*) Phylogenetic tree of *Caenorhabditis* species used in alignments. (*Right*) Splicing events were separated into three classes of exon triplets: constitutive exons, non-tissue-regulated alternative exons, and tissue-regulated alternative exons. Table shows number of exon triplets in each group and their conservation across the phylogeny. (*B*) Graph of percentage of conserved exon triplets for non-tissue-differential alternative exons (light blue bars) and tissue-differential alternative exons (gold bars) between *C. elegans* and other *Caenorhabditis* species of varying divergence. (*) *P*-value < 0.02, Fisher's exact test. (*C*) Plot of average phyloP score (rolling 3-nt average) over first 23 nt (*left*) and last 23 nt (*right*) of the upstream intron flanking the internal exon of the exon triplets described in panel *A*. (*D*) Same as in *C*, but plots of the average phyloP score for the downstream intronic regions.

Previous studies of tissue-regulated exons in vertebrates have shown that sequences flanking these exons tend to be more highly conserved (Sugnet et al. 2006; Fagnani et al. 2007). Thus, we next assessed whether tissue-regulated exons and their flanking introns and exons (exon triplets) were more likely to be conserved across the phylogeny of several *Caenorhabditis* species (Fig. 5A). For this analysis, we focused on comparing all cassette-type tissue-regulated exons with two other groups of exon triplets (composed of three exons and two introns). One group consisted of constitutively spliced events, in which three sequential exons are always spliced into mRNA transcripts. The other group consisted of non-tissue-regulated alternative exons and their flanking sequences, for which our TRAP-seq measurements suggest the alternative exons show little or no tissue-specific splicing differences. Collectively, our analysis included 113 tissue-regulated exons, 277 non-tissue-regulated exons, and 467 constitutive exons for comparison (for details, see Methods) (Fig. 5A).

Multiple sequence alignments of *C. elegans* exon triplets were performed with syntenic sequences from six species in the *Caenorhabditis* genus: *Caenorhabditis briggsae*, *Caenorhabditis remanei*, *Caenorhabditis tropicalis*, *Caenorhabditis brenneri*, *Caenorhabditis japonica*, and *Caenorhabditis angaria* (Fig. 5A). First, we investigated the degree of conservation of exon triplets from each group of splicing events, defined by the presence of all three exons and alignment of the splice sites surrounding these exons. As expected, we see a decrease in the number of conserved exon triplets as evolutionary divergence from *C. elegans* increases (Fig. 5A). We next calculated the percentage of conservation of exon triplets between *C. elegans* and the other species, excluding genes that had no clear homologous sequence alignments over any part of their coding region (see Methods). Extending earlier comparisons with *C. briggsae* and *C. remanei* using EST/cDNA data (Irimia et al. 2008), nearly two-thirds of all alternative exons are conserved up to the common ancestor of *C. elegans* and *C. japonica* (Fig. 5B). However, conservation of both classes of alternative exon triplets dropped substantially to ∼30% in alignments between the *C. elegans* and *C. angaria* genomes (Fig. 5B). Tissue-regulated alternative exons and their neighboring exons were found to be conserved at a modest, but consistently higher rate throughout the phylogeny compared with non-tissue-regulated alternative exons (Fig. 5B). In particular, *C. japonica* has a significantly greater proportion of conserved tissue-regulated exon triplets with *C. elegans* compared with non-tissue-regulated alternative splicing events (72% vs. 59%, respectively; *P*-value = 0.02, Fisher's exact test) (Fig. 5B).

Alternative splicing is known to be regulated by *cis* elements in exonic and intronic regions. As such, selection pressure to preserve these elements would increase conservation in the intronic regions surrounding alternative exons compared with analogous regions flanking constitutive exons. We investigated this characteristic by measuring average conservation in introns on an individual nucleotide level across the seven species described above using the program phyloP (Pollard et al. 2010). We focused on the 23 nt adjacent to each splice site, because this length would still partition our smallest introns (∼40 nt in length) into 5′ and 3′ halves. By comparing phyloP scores of 23 nt adjacent to each splice site, we observed increased sequence conservation surrounding alternative exons compared with constitutive exons (Fig. 5C,D). This increased conservation was particularly apparent in the splice sites and neighboring regions immediately adjacent to the alternative exons (comparing constitutive exons with both classes of alternative exons, at 3′ end of upstream intron or 5′ end of downstream intron; *P*-values < 2.2 × 10^−16^, Wilcoxon signed-rank test) (Fig. 5C,D). Moreover, the intronic regions immediately flanking tissue-regulated alternative exons were significantly more conserved than the corresponding sequences flanking non-tissue-regulated exons (3′ end of upstream intron, *P*-value < 2 × 10^−6^; 5′ end of downstream intron, *P*-value < 2.2 × 10^−16^; Wilcoxon signed-rank test) (Fig. 5C,D).

Taken together, these data suggest that the intronic regions surrounding alternative exons are considerably more conserved than analogous sequences in constitutive exons. Moreover, the extended conservation required for regulation of tissue-specific exons likely creates selection pressure to preserve neighboring exon–intron architecture across evolutionary timescales.

### Alternative and tissue-regulated exons are enriched in motifs recognized by RBPs

**Figure 6.**
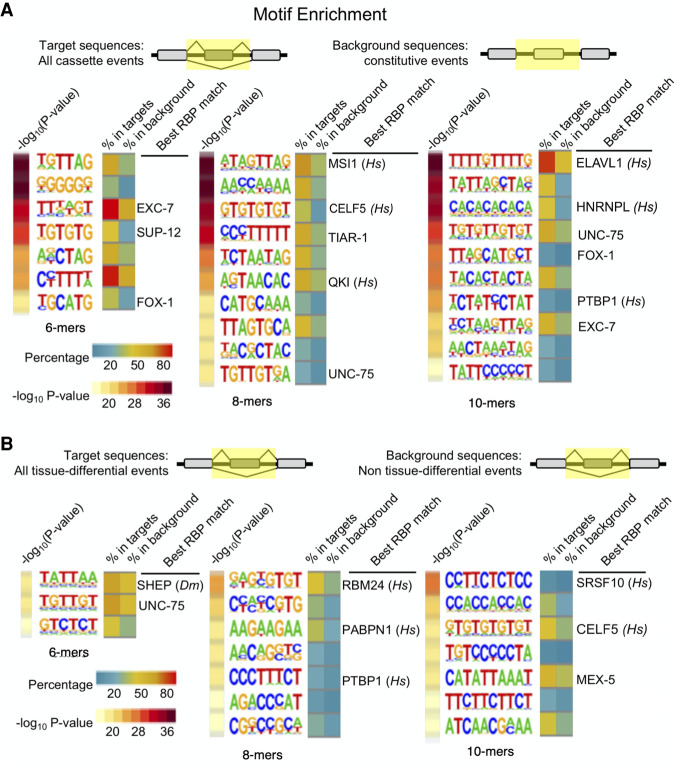
Enriched *cis* elements surrounding alternative exons and tissue-regulated alternative exons. (*A*) Enriched motifs identified when comparing all cassette-type alternative exons with constitutive exons. *Top* panel displays the regions compared (highlighted in yellow) spanning internal exon and flanking introns. Enrichment *P*-values are listed to the *left* of the motifs as −log_10_-transformed values (yellow–red scale). Motifs are presented as sequence logos derived from *cis* elements observed in the data. The percentage of sequences in the target group versus the background group are displayed to the *right* of the motif logos (blue–yellow–red scale). If a sequence motif overlaps with a known consensus motif for an RBP identified, the RBP is labeled as well as the species the consensus was derived from. (*Hs*) *Homo sapiens*. (*B*) Same as in *A*, except target sequences are tissue-regulated alternative splicing events and background sequences are non-tissue-regulated alternative splicing events. (*Hs*) *Homo sapiens*, (*Dm*) *D. melanogaster*.

Previous studies in *C. elegans* have shown that sequence enrichment search strategies can be effective at identifying candidate splicing regulatory signals (Kabat et al. 2006; Ramani et al. 2011). We used hypergeometric optimization of motif enrichment (HOMER) (Heinz et al. 2010), which can detect enriched motifs in a given list of sequences relative to a background list of sequences. We first compared sequences spanning alternative cassette exons and flanking introns to equivalent sequences surrounding constitutively included exons (Fig. 6A). The resulting enriched sequences yield several expected motifs previously identified (Kabat et al. 2006; Ramani et al. 2011) and recognized by characterized RBPs, including FOX-1/Rbfox and the muscle-specific regulator SUP-12 (an ortholog of Rbm24 and Rbm38), which have both been implicated in alternative splicing regulation in *C. elegans* (Kuroyanagi et al. 2007). We also identified enriched *cis* elements recognized by UNC-75/CELF and EXC-7 (an ortholog of Elavl4/HuD), both known to regulate tissue-specific alternative splicing within the nervous system (Kuroyanagi et al. 2013a,b; Norris et al. 2014; Chen et al. 2016).

We next searched for motifs enriched in tissue-regulated alternative splicing events relative to analogous sequences spanning non-tissue-regulated splicing events (Fig. 6B). Again, we identified motifs for known RBPs and splicing regulators. Some of these motifs were not identified in our alternative versus constitutive exon comparison above, suggesting that they are selectively associated with tissue-regulated splicing patterns.

In both comparisons, we also found enrichment of many motifs that could not be connected with a cognate RBP, as well as motifs that matched consensus sequences from other species (Fig. 6A,B). Taken together, our results indicate that alternative exons and, by extension, tissue-regulated exons, are coordinately regulated by RBPs, several of which remain to be discovered and characterized.

### UNC-75/CELF recognizes conserved motifs overlapping a 5′ splice site and represses exon inclusion

Both our conservation analysis (Fig. 5) and motif enrichment analysis (Fig. 6) indicated that tissue-regulated exons are flanked by conserved and overrepresented sequence features, likely reflecting the presence of critical *cis* elements. As such, we next assessed whether these analyses could prove useful in uncovering novel splicing regulatory mechanisms.

**Figure 7.**
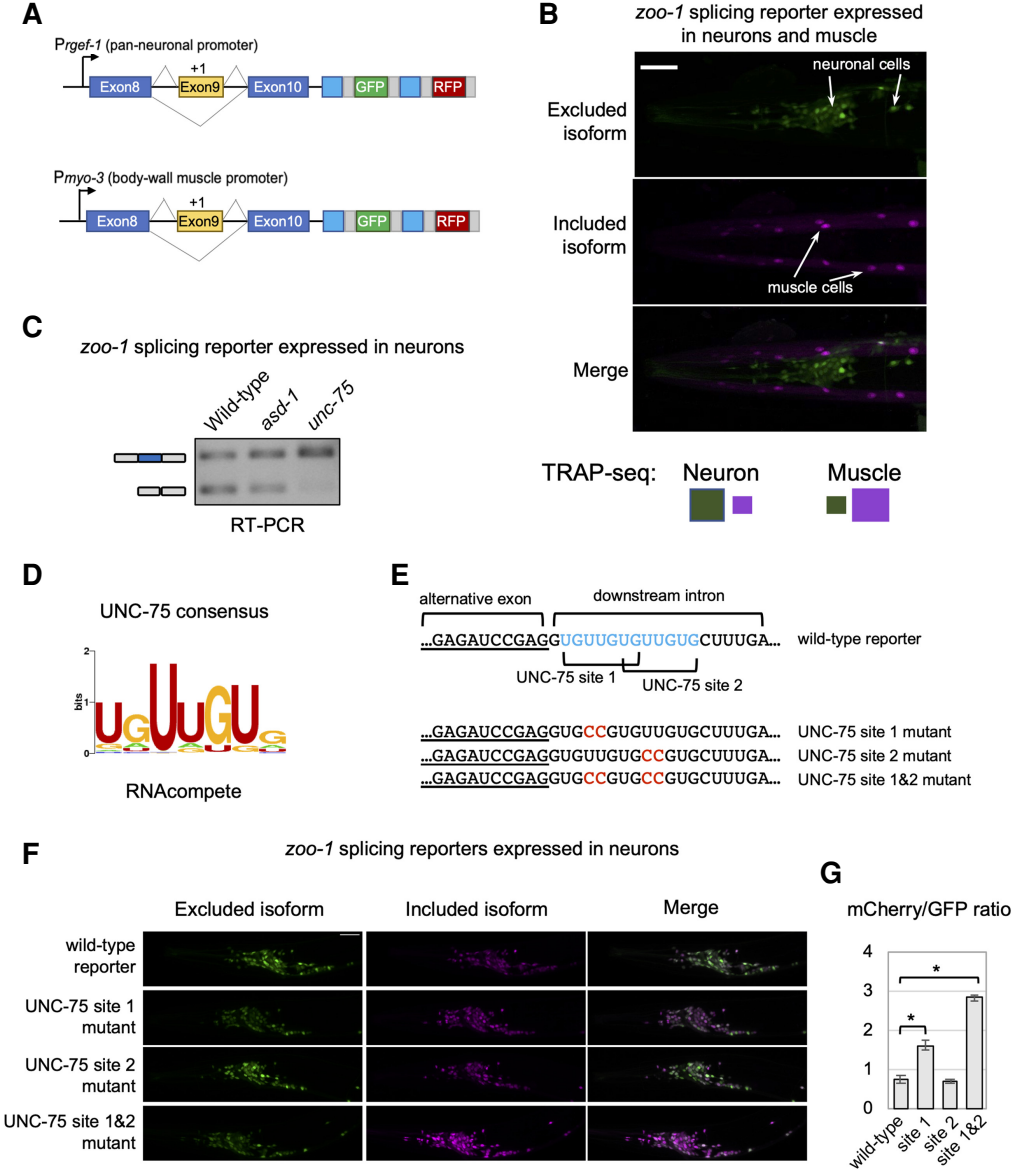
UNC-75/CELF represses inclusion of an exon by binding to *cis* elements overlapping a 5′ splice site. (*A*) Schematic of two-color reporters used for experiments. (P*rgef-1*) Promoter driving pan-neuronal expression; (P*myo-3*) promoter driving body-wall muscle expression. (*B*) Fluorescence microscopy images of *zoo-1* two-color reporter monitoring splicing of exon 9 in both neurons and muscle cells. Neurons display increased exon skipping (green), whereas muscle cells primarily include the alternative exon (purple). Scale bar, 20 μm. (*C*) RT-PCR assay monitoring *zoo-1* two-color splicing reporter expressed only in neurons in wild-type animals (lane *1*), *asd-1* mutants (lane *2*), and *unc-75* mutants (lane *3*). Primers are designed to amplify both exon 9 included (*top* band) and excluded (*bottom* band) isoforms. (*D*, *left*) UNC-75 consensus motif derived from RNAcompete data (Ray et al. 2013). (*E*, *top*) *S*chematic of 3′ end of *zoo-1* exon 9 (underlined) and flanking downstream intron sequence. Two UNC-75 consensus sequences overlapping the 5′ splice site are highlighted in blue. (*Bottom*) Reporters with mutated UNC-75 consensus sequences (substitutions in red). (*F*) Fluorescence microscopy images of wild-type and mutant two-color *zoo-1* reporters as described in *E*. Scale bar, 20 μm. (*G*) Bar graph displaying mCherry/GFP fluorescence intensity ratios for each *zoo-1* splicing reporter. Each bar represents mean ratio from five individual animals. Error bars, ± 1 SD from the mean value. (*) *P*-value < 0.01, Kruskal–Wallis rank sum test.

We identified conserved UNC-75 consensus sequences overlapping the 5′ splice site in the intron downstream from alternative exon 9 of the *zoo-1* gene (Fig. 7; Supplemental Fig. S5). This gene encodes an ortholog of the zonula occludens tight junctional protein ZO-2, a key protein involved in cell adhesion and cell proliferation (González-Mariscal et al. 2019). Our TRAP-seq data identified that *zoo-1* exon 9 is primarily excluded in neurons and mostly included in muscle cells (Fig. 7A). We further validated these patterns by coexpressing neuron and muscle promoter driven *zoo-1* two-color splicing reporters (Fig. 7A). Consistent with our TRAP-seq measurements, when signal from both cell types are imaged together, we detected significantly preferential inclusion of exon 9 (mCherry) in muscle cells and more biased skipping of exon 9 (GFP) in neuronal cells (Fig. 7B).

Given that UNC-75 is broadly expressed in the nervous system (Loria et al. 2003), we speculated that it has a direct role in blocking exon 9 inclusion through interaction with its cognate *cis* elements. To test this hypothesis, we expressed a *zoo-1* exon 9 splicing reporter in the nervous system of wild-type animals, animals lacking *unc-75*, or animals lacking *asd-1*, an unrelated RBP that is also expressed in the nervous system. RT-PCR assays amplifying both *zoo-1* isoforms were then performed on total RNA collected from these animals (Fig. 7C). Consistent with our prediction, exon 9 is more highly included in the neurons of *unc-75* loss-of-function mutants compared with wild-type animals (Fig. 7C). Mutants lacking *asd-1* had splicing patterns similar to wild-type animals, suggesting that loss of *unc-75* has a specific effect on *zoo-1* exon 9 alternative splicing (Fig. 7C). We next generated two-color splicing reporters with mutations targeting one or both UNC-75 consensus sequences (Fig. 7D,E) and drove expression of these reporters specifically in neurons (Fig. 7F). When imaging the neuron-expressed reporters in the absence of any muscle signal, the included isoform can now be detected with imaging settings that would otherwise saturate signal in muscle cells. We next quantified the total fluorescence signal through the head regions of animals expressing wild-type and mutant reporters (Fig. 7F,G). These experiments showed that mutations in the first or second *cis* elements increased the relative proportion of the included isoform in neurons (Fig. 7F,G). However, mutations targeting both *cis* elements led to the maximum effect on splicing, in which exon 9 is significantly more included in most neurons (*P*-value < 0.01, Kruskal-Wallis rank sum test) (Fig. 7F,G).

Taken together, these results indicate that CELF proteins can act as repressors of alternative exon inclusion in the nervous system when bound to *cis* elements directly overlapping with 5′ splice sites.

### Alternative exons and tissue-biased exons have distinct regulatory features compared with constitutive exons

Previous studies of vertebrate alternatively spliced exons and their flanking introns and exons have indicated that regulated exons have distinct sequence characteristics compared with analogous regions surrounding constitutively spliced exons (Xing and Lee 2005; Yeo et al. 2005; Yura et al. 2006; Keren et al. 2010; Barbosa-Morais et al. 2012). In a similar manner, we compared various sequence features among alternative exons, constitutive exons, and their surrounding sequences.

**Figure 8.**
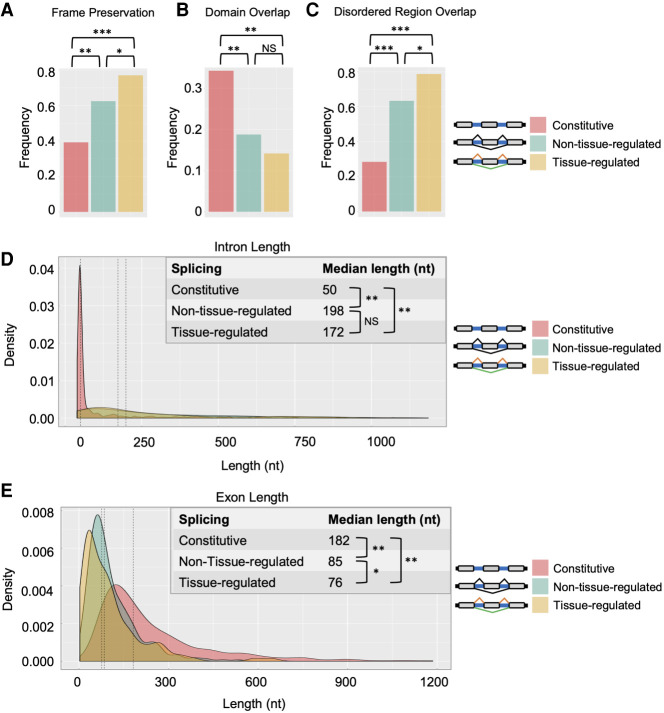
Features associated with alternative exons and tissue-regulated alternative exons. For panels *A*–*E*, comparisons were made between constitutively spliced exons (pink), alternative exons (light blue), and tissue-regulated alternative exons (gold). (*A*) Comparison of frequency of frame preservation (percentage of exons that are a multiple of 3 nt). (*B*) Frequency of exons overlapping with a UniProt conserved protein domains. (*C*) The frequency of exons overlapping with predicted disordered regions in proteins. (*D*) Distributions of the lengths of introns flanking internal exons from same comparison groups. (*E*) Distribution of the lengths of exons. (*A*,*C*) Pearson's chi-squared test: (*) *P* ≤ 0.01; (**) *P* < 1 × 10^−3^; (***) *P* < 1 × 10^−10^. (*D*,*E*) Wilcoxon rank-sum test: (*) *P* ≤ 0.05; (**) *P* < 1 × 10^−15^.

First, we tested the frequency of internal constitutive or alternative exons to preserve the open reading frame—specifically, if the exon is a multiple of 3 nt (Fig. 8A). Tissue-regulated alternative exons have been shown to have increased selection pressure to be frame-preserving in order to be included or skipped in transcripts without causing a shift in reading frame (Modrek et al. 2001). Consistent with these observations, we observe an increase in the frequency of alternative exons to preserve reading frame relative to constitutive exons (Fig. 8A). Frame preservation frequency is further increased among the tissue-regulated splicing events, in which ∼80% of exons are multiples of three.

We next tested the frequency with which internal constitutive or alternative exons overlap with conserved protein domains or intrinsically disordered regions (IDRs) (Fig. 8B,C). We found that alternative exons overlap less frequently with annotated protein domains (Fig. 8B) but more frequently overlap with IDRs, with tissue-regulated alternative exons having the highest degree of overlap with IDRs (approaching 80% of all exons) (Fig. 8C). Again, this trend is consistent with reports in vertebrates, where alternatively spliced exons are known to modulate protein–protein interactions and the formation of multivalent protein assemblies through altering IDR sequence features (Romero et al. 2006; Buljan et al. 2012; Ellis et al. 2012; Yang et al. 2016).

Finally, we measured the lengths of internal constitutive exons, alternative exons, and their flanking intronic regions (Fig. 8D,E). Consistent with previous observations (Fox-Walsh et al. 2005; Kabat et al. 2006; Kim et al. 2007), introns flanking both tissue-differential and non-tissue-differential alternative exons are significantly longer than introns flanking constitutively spliced exons (Fig. 8D). This tendency toward longer intron length is preserved in both upstream and downstream introns flanking alternative exons, with the downstream intron having a somewhat longer length (Supplemental Fig. S6). In contrast, we found that alternative exons are significantly smaller in length compared with constitutive exons (Fig. 8E). This tendency toward a shorter length was also more pronounced in the tissue-differential alternative exons.

Collectively, these results show that alternative exons and their flanking intronic sequences have features that distinguish them from constitutive exons. Moreover, these identified features show hallmarks shared by other invertebrate and vertebrate alternative exons, suggesting common evolutionary constraints and functional consequences of these exons across metazoans.

### Microexons are differentially regulated across cell types and highly conserved across *Caenorhabditis* species

**Figure 9.**
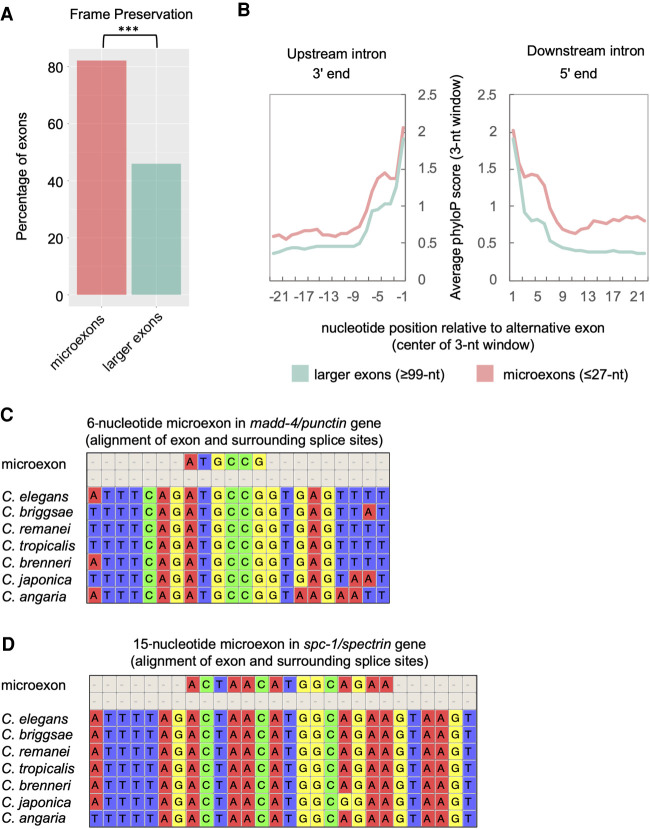
Microexons <27 nt are highly conserved in *Caenorhabditis* species. (*A*) Comparison of frame preservation frequency among microexons (pink) and larger exons (green). (***) *P* < 1 × 10^−10^, Pearson's chi-squared test. (*B*) Plot of average phyloP score (rolling 3-nt average) over last 23 nt (*left* panel) and first 23 nt (*right* panel) of the upstream and downstream introns flanking microexons (pink) or larger exons (green), respectively. (*C*) A multiple sequence alignment displaying a highly conserved 6-nt microexon and its surrounding splice sites in the *madd-4* gene. (*D*) A multiple sequence alignment of a highly conserved 15-nt microexon and its surrounding splice sites in the *spc-1* gene.

Our observations above indicated that tissue-regulated exons tend to be shorter than other classes of exons. Upon closer examination, we identified 24 microexons (≤27 nt in length) among these tissue-regulated exons (Fig. 9; Supplemental Table S7). Microexons have recently been identified as an interesting class of exons, with distinct mechanisms controlling their splicing patterns, and have shown roles in neuronal development and physiology in vertebrates (Scheckel and Darnell 2015). Moreover, the aberrant regulation of these exons has been associated with autism spectrum disorders (Irimia et al. 2014; Quesnel-Vallières et al. 2016; Gonatopoulos-Pournatzis et al. 2018).

Our identification of tissue-regulated microexons suggested that there may be additional microexons in our transcriptome data. We therefore slightly relaxed our filtering criteria and detected an additional 43 alternatively spliced microexons, for a total of 67 (Supplemental Table S7), indicating that this class of exons may be difficult to comprehensively detect in typical transcriptome analysis with short read data from whole-animal samples. Roughly one-third (23/67) microexons were detected exclusively in our tissue-enriched TRAP-seq data, suggesting that microexons often undergo tissue-differential splicing.

We next assessed whether microexons possessed features that distinguished them from larger alternative exons (≥99 nt). Microexons exhibit a high frequency of reading frame preservation (>80%) compared with larger exons (Fig. 9A). Additionally, an examination of intronic regions revealed that sequences surrounding microexons, particularly the 3′ end of the upstream intron and 5′ end of the downstream intron, are considerably more conserved than corresponding regions surrounding larger alternative exons (*P*-values all <1 × 10^−7^, Wilcoxon signed-rank test) (Fig. 9B). Representative multiple sequence alignments provide another view of this conservation. For example, the 6-nt microexon in the *madd-4/punctin* gene and the 15-nt microexon in the *spc-1* gene (a spectrin ortholog) were both found to show high levels of conservation within the exon and flanking intronic regions among all *Caenorhabditis* species examined (Fig. 9C,D).

Taken together, our analysis has shed light on an important class of exons that are likely influencing aspects of protein function and diversity.

## Discussion

In this study, we have highlighted the utility of the TRAP-seq approach for transcriptome-wide studies of features associated with alternative splicing across tissues and specific neuronal cell types. Our samples are also sequenced to fairly high depths of coverage (Supplemental Fig. S1), which has facilitated our ability to detect splice junctions with increased confidence. Consistent with this point, we down-sampled our short read data and showed that, although we are approaching saturation in our ability to detect alternatively spliced junctions (Supplemental Fig. S7), our current results are likely underestimating the full complexity of splicing in the isolated tissues of the organism. Our method provides a robust and complementary avenue for obtaining mRNA profiles along with other approaches such as mRNA tagging (Spencer et al. 2011; Blazie et al. 2015) and fluorescence-activated cell sorting of labeled cells (Spencer et al. 2014; Kaletsky et al. 2016).

Our results show that cells of the nervous system had the highest numbers of detected splice junctions (Fig. 1C), mirroring findings in vertebrates indicating that the nervous system exhibits relatively high levels of alternative splicing compared with other tissues (Barbosa-Morais et al. 2012; Tapial et al. 2017). It is only recently that neuronal-subtype differences in splicing have been interrogated and have revealed differences in isoform expression (Wamsley et al. 2018; Wang et al. 2018; Furlanis et al. 2019; Saito et al. 2019). Taken together, these observations and our own results suggest that an expanded use of alternative splicing is a hallmark of metazoan nervous systems, including the relatively compact nervous system of *C. elegans*.

Our observation that tissue-regulated splicing is enriched in complex events (Fig. 3A,B) in *C. elegans* is consistent with a recent study indicating that complex events account for a significant proportion of conserved differential splicing variation across mammalian tissues (Vaquero-Garcia et al. 2016). It is interesting to speculate how complex splicing events may arise during evolution. One likely model would involve complex events emerging from simpler ancestral splicing events. Comparisons of genomic and transcript sequences across multiple species may be able to provide some support of this model.

We identified highly conserved UNC-75/CELF binding sites overlapping directly with the 5′ splice site flanking alternative exon 9 of the *zoo-1* gene. We speculate a mechanism in which UNC-75 binds to these *cis* elements to repress inclusion of the alternative exon in neurons (Fig. 7). This mechanism of repression contrasts with the role of UNC-75 as an activator of exon inclusion when bound to a *cis* element 30 bases downstream from the 5′ splice site in the *unc-*16 gene (Norris et al. 2014). Recent studies have revealed position-dependent regulation of splicing by UNC-75 (Kuroyanagi et al. 2013b). Thus, small differences in the location and position of the UNC-75 *cis* elements can have distinct consequences on splicing regulation even within the same intronic region. It will be interesting to explore the biochemical mechanisms of UNC-75 binding to target mRNAs and investigate how prevalent this mode of CELF-mediated splicing repression is in multicellular animals.

Our results suggest that there are shared constraints across protein coding genes in multicellular animals that lead to the evolution of regulated splicing patterns. In particular, the possibility that tissue-regulated exons are more likely to be selected for influencing disordered regions in proteins is intriguing. Several emerging roles for disordered protein sequences include shaping protein–protein interactions and biophysical properties, as well as serving as sites of post-translational modifications (Wright and Dyson 2015). Given that tissue-regulated exons are highly conserved, their influence on protein function is likely important. An important goal will be to determine the molecular consequences of these splicing events on protein function in vivo.

Microexons have emerged as important modulators of protein function in vertebrates, particularly in the nervous system (Irimia et al. 2014; Li et al. 2015). Our current analysis has expanded the number of known microexons in *C. elegans*. Moreover, given that these microexons are highly conserved and often frame-preserving (Fig. 9), it is highly likely that these exons will influence protein function in different tissues, as recent studies in vertebrates have confirmed (Parras et al. 2018; Gonatopoulos-Pournatzis et al. 2020). It was proposed in a recent study that the explosion of microexons included in neuronal transcripts may have coincided with the evolution of the enhancer of microexons (eMIC) domain in the serine/arginine repetitive matrix (SRRM) protein family (Torres-Méndez et al. 2019). Other studies have also identified additional factors controlling the splicing of these exons (Li et al. 2015; Gonatopoulos-Pournatzis et al. 2018). However, SRRM proteins in *Caenorhabditis* species lack the eMIC domain (Torres-Méndez et al. 2019). Thus, *C. elegans* represents an interesting model system to explore ancient mechanisms governing microexon splicing.

Collectively, our work has revealed that alternative splicing represents a rich layer of gene expression, contributing to the specialized functions of cell and tissue types in *C. elegans*, particularly in the nervous system. As isoform-sensitive transcriptome profiling approaches at single-cell resolution are starting to emerge, we will likely gain an even deeper appreciation for the extent to which transcript diversity will ultimately impact the proteome and cellular specialization.

## Methods

### *C. elegans* maintenance and strains used in this study

Animals were maintained at 21°C and grown on nematode growth media plates seeded with OP50-1 bacteria under standard conditions (Brenner 1974). In addition to the N2 wild-type strain, the list of strains used in the current study is listed in Supplemental Table S8.

### TRAP-seq and read alignment

cDNA libraries from whole-animal input lysates and tissue-enriched data sets were created as described previously (Gracida et al. 2017). FASTQ files from paired-end Illumina sequencing data were used as input files, and reads were mapped using STAR aligner (Dobin et al. 2013), an algorithm capable of high-speed read alignment and splice junction detection. Genome index files were generated using *C. elegans* genome annotation WS251 and the default parameters. Two-pass read alignment was performed to increase detection of novel splice junctions. SAM and sorted BAM files were generated for subsequent analysis. For additional analysis to identify tissue- and neuron-subtype unique junction reads, see the Supplemental Material.

### Predicting tissue-differential splicing, categorizing splicing events into splicing classes, and characterization of splicing events

By using sorted BAM and BAM index files from our aligned reads, the Majiq/Voila software pipeline (Vaquero-Garcia et al. 2016) was applied to our TRAP-seq data to identify and visualize alternative splicing events (reported as local splicing variations [LSVs], within a single tissue) and differential splicing events (between two or more tissues). By using the output files of the Majiq analysis, more stringent filters were applied in order to obtain a set of high-confidence alternative splicing and differential splicing events for further computational analysis. All coding, statistical tests, and graphing were performed using R 3.5.1 (R Core Team 2018). For additional details, see Supplemental Material.

### Motif enrichment analysis

Motif analysis was performed using HOMER (Heinz et al. 2010) to discover annotated or de novo motifs that were enriched in tissue-regulated splicing events, using sets of constitutive splicing events or non-tissue-regulated splicing events as background sets. For specific details, see Supplemental Material.

### GO overrepresentation analysis

We used the online tool, WebGestalt (Liao et al. 2019) to perform the overrepresentation analyses. Sample data sets were (1) genes that are differentially spliced between any two tissues (ΔPSI ≥ 0.15), and (2) genes that are differentially expressed between any two tissues (with a 5× up- or down-regulated). In all comparisons, we search for enrichment against background gene sets in which genes were filtered for expression (above 50 read counts) within the tissue(s) involved. This filter controlled for simply enriching for genes that were differentially expressed in particular tissues. Overrepresentation analysis was performed to identify significantly enriched biological processes (Bonferroni multiple testing correction, corrected *P*-values < 0.05). This analysis was performed for differentially spliced/differentially expressed genes among the broad tissues and within the nervous system.

### Construction of two-color splicing reporter plasmids, microinjection, microscopy, and quantification

Our two-color reporters were generated using standard molecular biology approaches, integrated into animals, and visualized and quantified by confocal microscopy. For a list of primers used in the study, see Supplemental Table S9. For a list of plasmids used, see Supplemental Table S8. For additional details, please see Supplemental Material and Supplemental Figure S8.

### Multiple sequence alignments and analysis of sequence conservation

For multiple sequence alignments, we obtained coordinates of all relevant splicing events from our TRAP-seq data (spanning three exons and two introns, in which the internal exon is either an alternative or constitutive exon). By using these coordinates, corresponding BED files were generated and used to obtain multiple alignment format (MAF) files from the 26-way nematode alignment track on genome assembly ce11 (WBCel235) from the University of California Santa Cruz (UCSC) Genome Browser. We then extracted homologous sequences from seven species (*C. elegans*, *C. brenneri*, *C. briggsae*, *C. remanei*, *C. japonica*, *C. tropicalis*, and *C. angaria*) and realigned relevant regions surrounding alternative or constitutive exons using MUSCLE (Edgar 2004). To measure conservation patterns in a base-by-base manner, we used the program phyloP (Pollard et al. 2010), a part of the “rphast” R package (Hubisz et al. 2011). For additional details, see Supplemental Material.

## Data access

All raw and processed sequencing data generated in this study have been submitted to the NCBI Gene Expression Omnibus (GEO; https://www.ncbi.nlm.nih.gov/geo/) under accession number GSE106374.

## Competing interest statement

The authors declare no competing interests.

## Supplementary Material

Supplemental Material

## References

[GR267328KOTC1] Ahier A, Jarriault S. 2014 Simultaneous expression of multiple proteins under a single promoter in *Caenorhabditis elegans* via a versatile 2A-based toolkit. Genetics 196: 605–613. 10.1534/genetics.113.160846 24361941 PMC3948794

[GR267328KOTC2] Baeza-Centurion P, Miñana B, Schmiedel JM, Valcárcel J, Lehner B. 2019 Combinatorial genetics reveals a scaling law for the effects of mutations on splicing. Cell 176: 549–563.e23. 10.1016/j.cell.2018.12.010 30661752

[GR267328KOTC3] Barash Y, Calarco JA, Gao W, Pan Q, Wang X, Shai O, Blencowe BJ, Frey BJ. 2010 Deciphering the splicing code. Nature 465: 53–59. 10.1038/nature09000 20445623

[GR267328KOTC4] Barberan-Soler S, Zahler AM. 2008 Alternative splicing regulation during *C. elegans* development: splicing factors as regulated targets. PLoS Genet 4: e1000001 10.1371/journal.pgen.1000001 18454200 PMC2265522

[GR267328KOTC5] Barberan-Soler S, Lambert NJ, Zahler AM. 2009 Global analysis of alternative splicing uncovers developmental regulation of nonsense-mediated decay in *C. elegans*. RNA 15: 1652–1660. 10.1261/rna.1711109 19617316 PMC2743056

[GR267328KOTC6] Barbosa-Morais NL, Irimia M, Pan Q, Xiong HY, Gueroussov S, Lee LJ, Slobodeniuc V, Kutter C, Watt S, Çolak R, 2012 The evolutionary landscape of alternative splicing in vertebrate species. Science 338: 1587–1593. 10.1126/science.1230612 23258890

[GR267328KOTC7] Blazie SM, Babb C, Wilky H, Rawls A, Park JG, Mangone M. 2015 Comparative RNA-Seq analysis reveals pervasive tissue-specific alternative polyadenylation in *Caenorhabditis elegans* intestine and muscles. BMC Biol 13: 4 10.1186/s12915-015-0116-6 25601023 PMC4343181

[GR267328KOTC8] Blazie SM, Geissel HC, Wilky H, Joshi R, Newbern J, Mangone M. 2017 Alternative polyadenylation directs tissue-specific miRNA targeting in *Caenorhabditis elegans* somatic tissues. Genetics 206: 757–774. 10.1534/genetics.116.196774 28348061 PMC5499184

[GR267328KOTC9] Brenner S. 1974 The genetics of *Caenorhabditis elegans*. Genetics 77: 71–94. 4366476 10.1093/genetics/77.1.71 PMC1213120

[GR267328KOTC10] Brown JB, Boley N, Eisman R, May GE, Stoiber MH, Duff MO, Booth BW, Wen J, Park S, Suzuki AM, 2014 Diversity and dynamics of the *Drosophila* transcriptome. Nature 512: 393–399. 10.1038/nature12962 24670639 PMC4152413

[GR267328KOTC11] Buljan M, Chalancon G, Eustermann S, Wagner GP, Fuxreiter M, Bateman A, Babu MM. 2012 Tissue-specific splicing of disordered segments that embed binding motifs rewires protein interaction networks. Mol Cell 46: 871–883. 10.1016/j.molcel.2012.05.039 22749400 PMC3437557

[GR267328KOTC12] Chen L, Liu Z, Zhou B, Wei C, Zhou Y, Rosenfeld MG, Fu XD, Chisholm AD, Jin Y. 2016 CELF RNA binding proteins promote axon regeneration in *C. elegans* and mammals through alternative splicing of syntaxins. eLife 5: e16072 10.7554/eLife.16072 27253061 PMC4946901

[GR267328KOTC13] Dobin A, Davis CA, Schlesinger F, Drenkow J, Zaleski C, Jha S, Batut P, Chaisson M, Gingeras TR. 2013 STAR: ultrafast universal RNA-seq aligner. Bioinformatics 29: 15–21. 10.1093/bioinformatics/bts635 23104886 PMC3530905

[GR267328KOTC14] Edgar RC. 2004 MUSCLE: multiple sequence alignment with high accuracy and high throughput. Nucleic Acids Res 32: 1792–1797. 10.1093/nar/gkh340 15034147 PMC390337

[GR267328KOTC15] Ellis JD, Barrios-Rodiles M, Çolak R, Irimia M, Kim TH, Calarco JA, Wang X, Pan Q, O”Hanlon D, Kim PM, 2012 Tissue-specific alternative splicing remodels protein-protein interaction networks. Mol Cell 46: 884–892. 10.1016/j.molcel.2012.05.037 22749401

[GR267328KOTC16] Fagnani M, Barash Y, Ip JY, Misquitta C, Pan Q, Saltzman AL, Shai O, Lee L, Rozenhek A, Mohammad N, 2007 Functional coordination of alternative splicing in the mammalian central nervous system. Genome Biol 8: R108 10.1186/gb-2007-8-6-r108 17565696 PMC2394768

[GR267328KOTC17] Fox-Walsh KL, Dou Y, Lam BJ, Hung SP, Baldi PF, Hertel KJ. 2005 The architecture of pre-mRNAs affects mechanisms of splice-site pairing. Proc Natl Acad Sci 102: 16176–16181. 10.1073/pnas.0508489102 16260721 PMC1283478

[GR267328KOTC18] Fu XD, Ares M. 2014 Context-dependent control of alternative splicing by RNA-binding proteins. Nat Rev Genet 15: 689–701. 10.1038/nrg3778 25112293 PMC4440546

[GR267328KOTC19] Furlanis E, Traunmüller L, Fucile G, Scheiffele P. 2019 Landscape of ribosome-engaged transcript isoforms reveals extensive neuronal-cell-class-specific alternative splicing programs. Nat Neurosci 22: 1709–1717. 10.1038/s41593-019-0465-5 31451803 PMC6763336

[GR267328KOTC20] Gerstberger S, Hafner M, Tuschl T. 2014 A census of human RNA-binding proteins. Nat Rev Genet 15: 829–845. 10.1038/nrg3813 25365966 PMC11148870

[GR267328KOTC21] Gerstein MB, Lu ZJ, Van Nostrand EL, Cheng C, Arshinoff BI, Liu T, Yip KY, Robilotto R, Rechtsteiner A, Ikegami K, 2010 Integrative analysis of the *Caenorhabditis elegans* genome by the modENCODE project. Science 330: 1775–1787. 10.1126/science.1196914 21177976 PMC3142569

[GR267328KOTC22] Giudice J, Xia Z, Wang ET, Scavuzzo MA, Ward AJ, Kalsotra A, Wang W, Wehrens XHT, Burge CB, Li W, 2014 Alternative splicing regulates vesicular trafficking genes in cardiomyocytes during postnatal heart development. Nat Commun 5: 3603 10.1038/ncomms4603 24752171 PMC4018662

[GR267328KOTC23] Gonatopoulos-Pournatzis T, Wu M, Braunschweig U, Roth J, Han H, Best AJ, Raj B, Aregger M, O'Hanlon D, Ellis JD, 2018 Genome-wide CRISPR-Cas9 interrogation of splicing networks reveals a mechanism for recognition of autism-misregulated neuronal microexons. Mol Cell 72: 510-524.e12. 10.1016/j.molcel.2018.10.008 30388412

[GR267328KOTC24] Gonatopoulos-Pournatzis T, Niibori R, Salter EW, Weatheritt RJ, Tsang B, Farhangmehr S, Liang X, Braunschweig U, Roth J, Zhang S, 2020 Autism-misregulated eIF4G microexons control synaptic translation and higher order cognitive functions. Mol Cell 77: 1176–1192.e16. 10.1016/j.molcel.2020.01.006 31999954

[GR267328KOTC25] González-Mariscal L, Gallego-Gutiérrez H, González-González L, Hernández-Guzmán C. 2019 ZO-2 is a master regulator of gene expression, cell proliferation, cytoarchitecture, and cell size. Int J Mol Sci 20: 4128 10.3390/ijms20174128 PMC6747478 31450555

[GR267328KOTC26] Gracida X, Calarco JA. 2017 Cell type-specific transcriptome profiling in *C. elegans* using the translating ribosome affinity purification technique. Methods 126: 130–137. 10.1016/j.ymeth.2017.06.023 28648677

[GR267328KOTC27] Gracida X, Norris AD, Calarco JA. 2016 Regulation of tissue-specific alternative splicing: *C. elegans* as a model system. Adv Exp Med Biol 907: 229–261. 10.1007/978-3-319-29073-7_10 27256389

[GR267328KOTC28] Gracida X, Dion MF, Harris G, Zhang Y, Calarco JA. 2017 An Elongin-Cullin-SOCS Box complex regulates stress-induced serotonergic neuromodulation. Cell Rep 21: 3089–3101. 10.1016/j.celrep.2017.11.042 29241538 PMC6283282

[GR267328KOTC29] Heiman M, Schaefer A, Gong S, Peterson JD, Day M, Ramsey KE, Suárez-Fariñas M, Schwarz C, Stephan DA, Surmeier DJ, 2008 A translational profiling approach for the molecular characterization of CNS cell types. Cell 135: 738–748. 10.1016/j.cell.2008.10.028 19013281 PMC2696821

[GR267328KOTC30] Heinz S, Benner C, Spann N, Bertolino E, Lin YC, Laslo P, Cheng JX, Murre C, Singh H, Glass CK. 2010 Simple combinations of lineage-determining transcription factors prime *cis*-regulatory elements required for macrophage and B cell identities. Mol Cell 38: 576–589. 10.1016/j.molcel.2010.05.004 20513432 PMC2898526

[GR267328KOTC31] Hobert O. 2010 Neurogenesis in the nematode *Caenorhabditis elegans*. WormBook 1–24. 10.1895/wormbook.1.12.2 PMC4791530 20891032

[GR267328KOTC32] Hubisz MJ, Pollard KS, Siepel A. 2011 PHAST and RPHAST: phylogenetic analysis with space/time models. Brief Bioinform 12: 41–51. 10.1093/bib/bbq072 21278375 PMC3030812

[GR267328KOTC33] Irimia M, Rukov JL, Penny D, Garcia-Fernandez J, Vinther J, Roy SW. 2008 Widespread evolutionary conservation of alternatively spliced exons in *Caenorhabditis*. Mol Biol Evol 25: 375–382. 10.1093/molbev/msm262 18048400

[GR267328KOTC34] Irimia M, Weatheritt RJ, Ellis JD, Parikshak NN, Gonatopoulos-Pournatzis T, Babor M, Quesnel-Vallières M, Tapial J, Raj B, O'Hanlon D, 2014 A highly conserved program of neuronal microexons is misregulated in autistic brains. Cell 159: 1511–1523. 10.1016/j.cell.2014.11.035 25525873 PMC4390143

[GR267328KOTC35] Jaganathan K, Kyriazopoulou Panagiotopoulou S, McRae JF, Darbandi SF, Knowles D, Li YI, Kosmicki JA, Arbelaez J, Cui W, Schwartz GB, 2019 Predicting splicing from primary sequence with deep learning. Cell 176: 535-548.e24. 10.1016/j.cell.2018.12.015 30661751

[GR267328KOTC36] Kabat JL, Barberan-Soler S, McKenna P, Clawson H, Farrer T, Zahler AM. 2006 Intronic alternative splicing regulators identified by comparative genomics in nematodes. PLoS Comput Biol 2: e86 10.1371/journal.pcbi.0020086 16839192 PMC1500816

[GR267328KOTC37] Kaletsky R, Lakhina V, Arey R, Williams A, Landis J, Ashraf J, Murphy CT. 2016 The *C. elegans* adult neuronal IIS/FOXO transcriptome reveals adult phenotype regulators. Nature 529: 92–96. 10.1038/nature16483 26675724 PMC4708089

[GR267328KOTC38] Kaletsky R, Yao V, Williams A, Runnels AM, Tadych A, Zhou S, Troyanskaya OG, Murphy CT. 2018 Transcriptome analysis of adult *Caenorhabditis elegans* cells reveals tissue-specific gene and isoform expression. PLoS Genet 14: e1007559 10.1371/journal.pgen.1007559 30096138 PMC6105014

[GR267328KOTC39] Keren H, Lev-Maor G, Ast G. 2010 Alternative splicing and evolution: diversification, exon definition and function. Nat Rev Genet 11: 345–355. 10.1038/nrg2776 20376054

[GR267328KOTC40] Kim E, Magen A, Ast G. 2007 Different levels of alternative splicing among eukaryotes. Nucleic Acids Res 35: 125–131. 10.1093/nar/gkl924 17158149 PMC1802581

[GR267328KOTC41] Kotagama K, Schorr AL, Steber HS, Mangone M. 2019 ALG-1 influences accurate mrna splicing patterns in the *Caenorhabditis elegans* intestine and body muscle tissues by modulating splicing factor activities. Genetics 212: 931–951. 10.1534/genetics.119.302223 31073019 PMC6614907

[GR267328KOTC42] Kuroyanagi H, Ohno G, Mitani S, Hagiwara M. 2007 The Fox-1 family and SUP-12 coordinately regulate tissue-specific alternative splicing in vivo. Mol Cell Biol 27: 8612–8621. 10.1128/MCB.01508-07 17923701 PMC2169414

[GR267328KOTC43] Kuroyanagi H, Ohno G, Sakane H, Maruoka H, Hagiwara M. 2010 Visualization and genetic analysis of alternative splicing regulation in vivo using fluorescence reporters in transgenic *Caenorhabditis elegans*. Nat Protoc 5: 1495–1517. 10.1038/nprot.2010.107 20725066

[GR267328KOTC44] Kuroyanagi H, Watanabe Y, Hagiwara M. 2013a CELF family RNA-binding protein UNC-75 regulates Two sets of mutually exclusive exons of the unc-32 gene in neuron-specific manners in *Caenorhabditis elegans*. PLoS Genet 9: e1003337 10.1371/journal.pgen.1003337 23468662 PMC3585155

[GR267328KOTC45] Kuroyanagi H, Watanabe Y, Suzuki Y, Hagiwara M. 2013b Position-dependent and neuron-specific splicing regulation by the CELF family RNA-binding protein UNC-75 in *Caenorhabditis elegans*. Nucleic Acids Res 41: 4015–4025. 10.1093/nar/gkt097 23416545 PMC3627589

[GR267328KOTC46] Lee Y, Rio DC. 2015 Mechanisms and regulation of alternative pre-mRNA splicing. Annu Rev Biochem 84: 291–323. 10.1146/annurev-biochem-060614-034316 25784052 PMC4526142

[GR267328KOTC47] Li YI, Sanchez-Pulido L, Haerty W, Ponting CP. 2015 RBFOX and PTBP1 proteins regulate the alternative splicing of micro-exons in human brain transcripts. Genome Res 25: 1–13. 10.1101/gr.181990.114 PMC4317164 25524026

[GR267328KOTC48] Li R, Ren X, Ding Q, Bi Y, Xie D, Zhao Z. 2020 Direct full-length RNA sequencing reveals unexpected transcriptome complexity during *Caenorhabditis elegans* development. Genome Res 30: 287–298. 10.1101/gr.251512.119 32024662 PMC7050527

[GR267328KOTC49] Liao Y, Wang J, Jaehnig EJ, Shi Z, Zhang B. 2019 Webgestalt 2019: gene set analysis toolkit with revamped UIs and APIs. Nucleic Acids Res 47: W199–W205. 10.1093/nar/gkz401 31114916 PMC6602449

[GR267328KOTC50] Loria PM, Duke A, Rand JB, Hobert O. 2003 Two neuronal, nuclear-localized RNA binding proteins involved in synaptic transmission. Curr Biol 13: 1317–1323. 10.1016/S0960-9822(03)00532-3 12906792

[GR267328KOTC51] Lyssenko NN, Hanna-Rose W, Schlegel RA. 2007 Cognate putative nuclear localization signal effects strong nuclear localization of a GFP reporter and facilitates gene expression studies in *Caenorhabditis elegans*. BioTechniques 43: 596–600. 10.2144/000112615 18072588

[GR267328KOTC52] Merkin J, Russell C, Chen P, Burge CB. 2012 Evolutionary dynamics of gene and isoform regulation in mammalian tissues. Science 338: 1593–1599. 10.1126/science.1228186 23258891 PMC3568499

[GR267328KOTC53] Modrek B, Resch A, Grasso C, Lee C. 2001 Genome-wide detection of alternative splicing in expressed sequences of human genes. Nucleic Acids Res 29: 2850–2859. 10.1093/nar/29.13.2850 11433032 PMC55780

[GR267328KOTC54] Norris AD, Calarco JA. 2012 Emerging roles of alternative pre-mRNA splicing regulation in neuronal development and function. Front Neurosci 6: 122 10.3389/fnins.2012.00122 22936897 PMC3424503

[GR267328KOTC55] Norris AD, Gao S, Norris ML, Ray D, Ramani AK, Fraser AG, Morris Q, Hughes TR, Zhen M, Calarco JA. 2014 A pair of RNA-binding proteins controls networks of splicing events contributing to specialization of neural cell types. Mol Cell 54: 946–959. 10.1016/j.molcel.2014.05.004 24910101 PMC4096705

[GR267328KOTC56] Pan Q, Shai O, Misquitta C, Zhang W, Saltzman AL, Mohammad N, Babak T, Siu H, Hughes TR, Morris QD, 2004 Revealing global regulatory features of mammalian alternative splicing using a quantitative microarray platform. Mol Cell 16: 929–941. 10.1016/j.molcel.2004.12.004 15610736

[GR267328KOTC57] Pan Q, Shai O, Lee LJ, Frey BJ, Blencowe BJ. 2008 Deep surveying of alternative splicing complexity in the human transcriptome by high-throughput sequencing. Nat Genet 40: 1413–1415. 10.1038/ng.259 18978789

[GR267328KOTC58] Parras A, Anta H, Santos-Galindo M, Swarup V, Elorza A, Nieto-González JL, Picó S, Hernández IH, Díaz-Hernández JI, Belloc E, 2018 Autism-like phenotype and risk gene mRNA deadenylation by CPEB4 mis-splicing. Nature 560: 441–446. 10.1038/s41586-018-0423-5 30111840 PMC6217926

[GR267328KOTC59] Pollard KS, Hubisz MJ, Rosenbloom KR, Siepel A. 2010 Detection of nonneutral substitution rates on mammalian phylogenies. Genome Res 20: 110–121. 10.1101/gr.097857.109 19858363 PMC2798823

[GR267328KOTC60] Quesnel-Vallières M, Dargaei Z, Irimia M, Gonatopoulos-Pournatzis T, Ip JY, Wu M, Sterne-Weiler T, Nakagawa S, Woodin MA, Blencowe BJ, 2016 Misregulation of an activity-dependent splicing network as a common mechanism underlying autism spectrum disorders. Mol Cell 64: 1023–1034. 10.1016/j.molcel.2016.11.033 27984743

[GR267328KOTC61] Ragle JM, Katzman S, Akers TF, Barberan-Soler S, Zahler AM. 2015 Coordinated tissue-specific regulation of adjacent alternative 3′ splice sites in *C. elegans*. Genome Res 25: 982–994. 10.1101/gr.186783.114 25922281 PMC4484395

[GR267328KOTC62] Raj B, Blencowe BJ. 2015 Alternative splicing in the mammalian nervous system: recent insights into mechanisms and functional roles. Neuron 87: 14–27. 10.1016/j.neuron.2015.05.004 26139367

[GR267328KOTC63] Ramani AK, Calarco JA, Pan Q, Mavandadi S, Wang Y, Nelson AC, Lee LJ, Morris Q, Blencowe BJ, Zhen M, 2011 Genome-wide analysis of alternative splicing in *Caenorhabditis elegans*. Genome Res 21: 342–348. 10.1101/gr.114645.110 21177968 PMC3032936

[GR267328KOTC640] Ray D, Kazan H, Cook KB, Weirauch MT, Najafabadi HS, Li X, Gueroussov S, Albu M, Zheng H, Yang A, 2013 A compendium of RNA-binding motifs for decoding gene regulation. Nature 499: 172–177. 10.1038/nature12311 23846655 PMC3929597

[GR267328KOTC64] R Core Team. 2018 R: a language and environment for statistical computing. R Foundation for Statistical Computing, Vienna https://www.R-project.org/.

[GR267328KOTC65] Roach NP, Sadowski N, Alessi AF, Timp W, Taylor J, Kim JK. 2020 The full-length transcriptome of *C. elegans* using direct RNA sequencing. Genome Res 30: 299–312. 10.1101/gr.251314.119 32024661 PMC7050520

[GR267328KOTC66] Romero PR, Zaidi S, Fang YY, Uversky VN, Radivojac P, Oldfield CJ, Cortese MS, Sickmeier M, LeGall T, Obradovic Z, 2006 Alternative splicing in concert with protein intrinsic disorder enables increased functional diversity in multicellular organisms. Proc Natl Acad Sci 103: 8390–8395. 10.1073/pnas.0507916103 16717195 PMC1482503

[GR267328KOTC67] Saito Y, Yuan Y, Zucker-Scharff I, Fak JJ, Jereb S, Tajima Y, Licatalosi DD, Darnell RB. 2019 Differential NOVA2-mediated splicing in excitatory and inhibitory neurons regulates cortical development and cerebellar function. Neuron 101: 707-720.e5. 10.1016/j.neuron.2018.12.019 30638744 PMC6649687

[GR267328KOTC68] Scheckel C, Darnell RB. 2015 Microexons—tiny but mighty. EMBO J 34: 273–274. 10.15252/embj.201490651 25535247 PMC4339116

[GR267328KOTC69] Spencer WC, Zeller G, Watson JD, Henz SR, Watkins KL, McWhirter RD, Petersen S, Sreedharan VT, Widmer C, Jo J, 2011 A spatial and temporal map of *C. elegans* gene expression. Genome Res 21: 325–341. 10.1101/gr.114595.110 21177967 PMC3032935

[GR267328KOTC70] Spencer WC, McWhirter R, Miller T, Strasbourger P, Thompson O, Hillier LDW, Waterston RH, Miller DM. 2014 Isolation of specific neurons from C*. elegans* larvae for gene expression profiling. PLoS One 9: e112102 10.1371/journal.pone.0112102 25372608 PMC4221280

[GR267328KOTC71] Sugnet CW, Srinivasan K, Clark TA, O'Brien G, Cline MS, Wang H, Williams A, Kulp D, Blume JE, Haussler D, 2006 Unusual intron conservation near tissue-regulated exons found by splicing microarrays. PLoS Comput Biol 2: e4 10.1371/journal.pcbi.0020004 16424921 PMC1331982

[GR267328KOTC72] Tapial J, Ha KCH, Sterne-Weiler T, Gohr A, Braunschweig U, Hermoso-Pulido A, Quesnel-Vallières M, Permanyer J, Sodaei R, Marquez Y, 2017 An atlas of alternative splicing profiles and functional associations reveals new regulatory programs and genes that simultaneously express multiple major isoforms. Genome Res 27: 1759–1768. 10.1101/gr.220962.117 28855263 PMC5630039

[GR267328KOTC73] Torres-Méndez A, Bonnal S, Marquez Y, Roth J, Iglesias M, Permanyer J, Almudí I, O'Hanlon D, Guitart T, Soller M, 2019 A novel protein domain in an ancestral splicing factor drove the evolution of neural microexons. Nat Ecol Evol 3: 691–701. 10.1038/s41559-019-0813-6 30833759

[GR267328KOTC74] Tourasse NJ, Millet JRM, Dupuy D. 2017 Quantitative RNA-seq meta-analysis of alternative exon usage in *C. elegans*. Genome Res 27: 2120–2128. 10.1101/gr.224626.117 29089372 PMC5741048

[GR267328KOTC75] Ule J, Darnell RB. 2006 RNA binding proteins and the regulation of neuronal synaptic plasticity. Curr Opin Neurobiol 16: 102–110. 10.1016/j.conb.2006.01.003 16418001

[GR267328KOTC76] Vaquero-Garcia J, Barrera A, Gazzara MR, González-Vallinas J, Lahens NF, Hogenesch JB, Lynch KW, Barash Y. 2016 A new view of transcriptome complexity and regulation through the lens of local splicing variations. eLife 5: e11752 10.7554/eLife.11752 26829591 PMC4801060

[GR267328KOTC77] Vuong CK, Black DL, Zheng S. 2016 The neurogenetics of alternative splicing. Nat Rev Neurosci 17: 265–281. 10.1038/nrn.2016.27 27094079 PMC4861142

[GR267328KOTC78] Wamsley B, Jaglin XH, Favuzzi E, Quattrocolo G, Nigro MJ, Yusuf N, Khodadadi-Jamayran A, Rudy B, Fishell G. 2018 Rbfox1 mediates cell-type-specific splicing in cortical interneurons. Neuron 100: 846-859.e7. 10.1016/j.neuron.2018.09.026 30318414 PMC6541232

[GR267328KOTC79] Wang Z, Burge CB. 2008 Splicing regulation: from a parts list of regulatory elements to an integrated splicing code. RNA 14: 802–813. 10.1261/rna.876308 18369186 PMC2327353

[GR267328KOTC80] Wang ET, Sandberg R, Luo S, Khrebtukova I, Zhang L, Mayr C, Kingsmore SF, Schroth GP, Burge CB. 2008 Alternative isoform regulation in human tissue transcriptomes. Nature 456: 470–476. 10.1038/nature07509 18978772 PMC2593745

[GR267328KOTC81] Wang Q, Abruzzi KC, Rosbash M, Rio DC. 2018 Striking circadian neuron diversity and cycling of drosophila alternative splicing. eLife 7: e35618 10.7554/eLife.35618 29863472 PMC6025963

[GR267328KOTC82] Wani S, Kuroyanagi H. 2017 An emerging model organism *Caenorhabditis elegans* for alternative pre-mRNA processing in vivo. Wiley Interdiscip Rev RNA 8 10.1002/wrna.1428 28703462

[GR267328KOTC83] Warner AD, Gevirtzman L, Hillier LDW, Ewing B, Waterston RH. 2019 The *C. elegans* embryonic transcriptome with tissue, time, and alternative splicing resolution. Genome Res 29: 1036–1045. 10.1101/gr.243394.118 31123079 PMC6581053

[GR267328KOTC84] White JG, Southgate E, Thomson JN, Brenner S. 1986 The structure of the nervous system of the nematode *Caenorhabditis elegans*. Philos Trans R Soc London B, Biol Sci 314: 1–340. 10.1098/rstb.1986.0056 22462104

[GR267328KOTC85] Wright PE, Dyson HJ. 2015 Intrinsically disordered proteins in cellular signalling and regulation. Nat Rev Mol Cell Biol 16: 18–29. 10.1038/nrm3920 25531225 PMC4405151

[GR267328KOTC86] Xing Y, Lee CJ. 2005 Protein modularity of alternatively spliced exons is associated with tissue-specific regulation of alternative splicing. PLoS Genet 1: e34 10.1371/journal.pgen.0010034 16170410 PMC1201369

[GR267328KOTC87] Xiong HY, Alipanahi B, Lee LJ, Bretschneider H, Merico D, Yuen RKC, Hua Y, Gueroussov S, Najafabadi HS, Hughes TR, 2015 The human splicing code reveals new insights into the genetic determinants of disease. Science 347: 1254806 10.1126/science.1254806 25525159 PMC4362528

[GR267328KOTC88] Xu Q, Modrek B, Lee C. 2002 Genome-wide detection of tissue-specific alternative splicing in the human transcriptome. Nucleic Acids Res 30: 3754–3766. 10.1093/nar/gkf492 12202761 PMC137414

[GR267328KOTC89] Yang X, Coulombe-Huntington J, Kang S, Sheynkman GM, Hao T, Richardson A, Sun S, Yang F, Shen YA, Murray RR, 2016 Widespread expansion of protein interaction capabilities by alternative splicing. Cell 164: 805–817. 10.1016/j.cell.2016.01.029 26871637 PMC4882190

[GR267328KOTC90] Yeo G, Holste D, Kreiman G, Burge CB. 2004 Variation in alternative splicing across human tissues. Genome Biol 5: R74 10.1186/gb-2004-5-10-r74 15461793 PMC545594

[GR267328KOTC91] Yeo GW, Van Nostrand E, Holste D, Poggio T, Burge CB. 2005 Identification and analysis of alternative splicing events conserved in human and mouse. Proc Natl Acad Sci 102: 2850–2855. 10.1073/pnas.0409742102 15708978 PMC548664

[GR267328KOTC92] Yura K, Shionyu M, Hagino K, Hijikata A, Hirashima Y, Nakahara T, Eguchi T, Shinoda K, Yamaguchi A, Takahashi K-i, 2006 Alternative splicing in human transcriptome: functional and structural influence on proteins. Gene 380: 63–71. 10.1016/j.gene.2006.05.015 16872759

[GR267328KOTC93] Zahler AM. 2012 Pre-mRNA splicing and its regulation in *Caenorhabditis elegans*. WormBook 1–21. 10.1895/wormbook.1.31.2 PMC4781317 22467343

[GR267328KOTC94] Zhang C, Frias MA, Mele A, Ruggiu M, Eom T, Marney CB, Wang H, Licatalosi DD, Fak JJ, Darnell RB. 2010 Integrative modeling defines the nova splicing-regulatory network and its combinatorial controls. Science 329: 439–443. 10.1126/science.1191150 20558669 PMC3412410

[GR267328KOTC95] Zheng S, Black DL. 2013 Alternative pre-mRNA splicing in neurons: growing up and extending its reach. Trends Genet 29: 442–448. 10.1016/j.tig.2013.04.003 23648015 PMC3959871

