# Supplementary material for: Global regulatory features of alternative splicing across tissues and within the nervous system of C. elegans
Source: Genome Res. 2020 Dec;30(12):1766–80. doi: 10.1101/gr.267328.120 (PMC7706725; doi:10.1101/gr.267328.120)
Supplement: Supplemental Material [file supp_30_12_1766__index.html]

Global regulatory features of alternative splicing across tissues and within the nervous system of C. elegans — Supplemental Material 

# Global regulatory features of alternative splicing across tissues and within the nervous system of *C. elegans*

## Supplemental Material

- Supplemental\_Material.pdf
- Supplemental\_Table\_S1.xlsx
- Supplemental\_Table\_S2.xlsx
- Supplemental\_Table\_S3.xlsx
- Supplemental\_Table\_S4.xlsx
- Supplemental\_Table\_S5.xlsx
- Supplemental\_Table\_S6.xlsx
- Supplemental\_Table\_S7.xlsx
- Supplemental\_Table\_S8.xlsx
- Supplemental\_Table\_S9.xlsx
