# Supplementary material for: Global regulatory features of alternative splicing across tissues and within the nervous system of C. elegans
Source: Genome Res. 2020 Dec;30(12):1766–80. doi: 10.1101/gr.267328.120 (PMC7706725; doi:10.1101/gr.267328.120)
Supplement: Supplemental Material [file supp_gr.267328.120_Supplemental_Material.pdf]

Supplemental Material For:

Global regulatory features of alternative splicing across tissues and within the nervous system of *C. elegans*

Table of Contents:

**Supplemental Methods**	**Page 2**
**Supplemental References**	**Page 12**
**Supplemental Figs 1-8**	**Page 14**

Supplemental Methods

Tissue- and neuron-subtype unique reads absent in whole-animal input samples

All input control RNA-seq reads were merged (from ten files) and both replicates of each tissue- and neuron-subtype were also merged to create summed read and splice junction count files for each representative sample. Junction counts were obtained from the SJ.out files generated by STAR alignment. Coordinates of splice junctions present in tissue- and neuron-subtype samples were compared against junctions detected in input, and any junctions that were absent (had 0 counts) in all of the input samples, were classified as 'tissue unique' junctions. These junctions were then filtered according to read coverage, requiring coverage by a minimum of five, ten, fifteen or twenty reads.

Junction usage of tissue- and neuron-subtype unique reads

Relative splice junction usage was calculated with an approach similar to (Tourasse et al. 2017). All junctions for which start and end coordinates fell within the boundaries of a gene on the same strand were assigned to that gene. Gene boundaries were compiled with genome annotation cel235.92 (GTF file obtained from Ensembl database) as well as the most updated gene predictions for each chromosome. Gene start and end coordinates were denoted as the beginning of the first exon the end of the last exon, respectively. For each splice junction within a given gene, a donor site and acceptor site ratio were calculated by dividing the number of reads for that junction by the summed total of mapped reads from all the splice junctions that share that donor site or acceptor site.

Several cases were considered for calculating the splice junction usage ratio. In the case that only one of the two donor or acceptor sites are shared by other splice junctions, the usage ratio calculated for the site that is shared is kept as the representing ratio. In the case that both the donor site and the acceptor site of a particular splice junction are shared by multiple splice junctions and two usage ratios can be calculated, the usage ratio calculated from the donor/acceptor site with the largest number of reads is kept. Lastly, in the case where a splice junction neither shares the donor nor the acceptor site with other splice junctions, a ratio of one is assigned to that splice junction, and it is considered constitutively spliced. In this way, all of the junction usage ratios were calculated for input control and compared with the ratios recorded in a recent splice junction meta-analysis study—we refer to this dataset as the "RNA-seq compendium" (Tourasse et al. 2017). The Pearson correlation coefficient between the input control and RNA-seq compendium junction usage ratios was measured in R 3.5.0 (3.5.1. 2018). Junction usage ratios were also calculated for the 'tissue unique' junctions from the broad tissue and neuron-subtype RNA-seq reads. This list was subsequently filtered to remove junctions that have fewer that 20 supporting reads and/or show constitutive splicing in every tissue. The remaining splice junctions were compared to the junctions from the RNA-seq compendium.

Additional filters for stringency and reproducibility to generate high-confidence lists of alternative splicing events

Alternative splicing analysis: The Percent Spliced In (PSI) values are a measure of the frequency of reads supporting a particular junction relative to the other junctions that share either the same donor site or acceptor site; in Majiq this group of junctions is referred to as an LSV. We required that PSI values for each junction were ≥ 5% and ≤ 95% to be considered 'alternative'. Splice

junction output files from the STAR analysis were retrieved for each replicate and LSVs with at least 20 summed reads supporting the junctions composing that LSV were retained. To ensure reproducibility, alternative splicing events were also required to have a maximum difference in PSI (ΔPSI) value of ≤ 25% in both biological replicates.

Tissue-differential splicing analysis: ΔPSI values are measures of the difference in the frequency of a junction between two different tissue types. ΔPSI values were computed by Majiq for six pairwise tissue comparisons that were performed: muscle vs. intestine, nervous system vs. muscle, nervous system vs. intestine, nervous system vs. dopaminergic neuron subtype, nervous system vs. serotonergic neuron subtype and dopaminergic vs. serotonergic neuron subtypes. Again, to apply more stringent parameters, a minimum PSI value of 0.05 was required for both of the two tissues in the comparison. As stated above, LSVs must have a minimum of 20 supporting reads summed for all the junctions in each replicate. The ΔPSI values for each pairwise tissue comparison were calculated for each biological replicate and the magnitude of each ΔPSI value was required to be ≥0.15 and the difference in ΔPSI values between replicates was required to be <0.1.

An identical approach was used to classify alternative and tissue-differential splicing events into the major splicing classes: cassette-type, alternative 3'/5' splice site selection, alternative start/terminal exons, mutually exclusive exons and intron retention. Majiq details the coordinates of the junctions and exon that are in each LSV and each splicing class can be discriminated by using this information (see Voila splice graphs, Fig S3). Cassette-type exons will have two LSVs with two junctions each, involving the same three exons; one junction unique to each LSV and one shared. Alternative 3'/5' splice site selection will have one LSV comprised of two junctions and

two exons. Similarly, alternative start or terminal exons will have two junctions, both with the same donor site on a common exon, and two more exons that have no upstream or downstream junction connectivity, respectively. Mutually exclusive exons have two LSVs with two junctions and three exons each. The two exons that are shared by both LSVs are the mutually exclusive exons, which have no connecting junction. Intron retention events were explicitly detected by Majiq and filtered for sufficient read coverage and reproducibility. Complex events were classified as LSVs with more than three junctions connecting more than four exons. Lastly, for the splicing events of all classes, it was verified that no junctions external to the relevant LSVs overlapped within the boundaries of each categorized splicing event.

The elevated numbers of alternative splice site usage relative to other classes of splicing has been an interesting observation that we have observed in a previous study (Ramani et al. 2011). We suspect that alternative splice site usage may have evolved to be more commonplace in nematodes, although the reason for this is unclear. It is possible that the prevalence of detected alternative splice site selection events is due to poor sequence depth at one side of a *bona fide* alternative cassette exon. However, we have applied a number of filters (including a threshold for the minimum number of junction counts to support an alternative splicing event) in an attempt to minimize such mis-annotations. Although we cannot completely rule out this possibility, it is most likely that in cases where there is sufficient coverage and yet only two out of three junctions are detected, the event in question is a *bona fide* alternative splice site selection event rather than a mis-annotated cassette event.

Microexon detection

For the genome-wide detection of microexons, a work-flow similar to the detection of cassette-type splicing events above was used. Cassette events from all input and tissue-specific transcriptomes were found that had a minimum PSI value of 0.05 and no minimum read count cutoff. Events were filtered for alternative exons that were ≤ 27 nucleotides.

Comparison of gene expression and junction usage

HTSeq (Anders et al. 2015) was used to obtain raw read counts mapping to each gene. All raw count values were then normalized using upper quartile normalization. Following normalization, all average TRAP sample to input control fold changes were computed for each gene. Every pairwise tissue comparison (as listed above) was filtered for at least one tissue having a minimum of 50 reads in the input or TRAP sample. Only genes that were 5x up-or down-regulated in expression between any two tissues were selected. The fold changes for each tissue were then log_2-transformed and centered around the median fold change among all three tissues in the comparison. Out of this list of genes, those that also contained alternative splicing events were selected and the PSI values observed for the most differentially spliced junction within this gene was obtained for each of the relevant tissue types. Clustering of gene expression patterns was performed with Cluster 3.0 (de Hoon et al. 2004) using average-linkage hierarchical clustering with Euclidean distance. Spearman's rank correlation coefficient was computed to assess the relationship between changes in gene expression and alternative splicing. Similarly, for the opposite analysis of tissue-differential alternative splicing vs gene expression, gene expression differences for genes containing junctions that are highly differentially spliced between two or more tissues (ΔPSI ≥0.15) were obtained as described above and clustering was instead performed for PSI values.

Characterizing tissue-differential splicing events

Three datasets were generated to explore various patterns in cassette-type splicing events. 1) Tissue-differential: cassette events in which the PSI value of the alternatively spliced exon differed significantly between two or more tissues. 2) Non-tissue-differential: events in which the PSI value of an alternatively spliced exon does not differ significantly between any tissues. 3) Constitutive splicing events: exons that are always included in the final mRNA transcript. For all three datasets, it was ensured that the upstream and downstream exons of the exon of interest were constitutively spliced. Coordinates and sequences of all upstream, middle and downstream exons and upstream and downstream introns were extracted for subsequent analysis.

These sets of exons and introns were used to calculate median size, frequency of the exons being a multiple of three nucleotides (frame preservation), frequency of overlap with a protein domain or overlap with a disordered domain. Middle exon coordinates were input as tracks into the UCSC Genome Browser (ce11) and overlapping UniProt domains were extracted using the Data Integrator tool. In order to test for exon overlap with disordered regions, protein sequences containing the middle exons of all three datasets was obtained from the WormMine data mining tool in WormBase (Grove et al. 2018). In the case of the tissue-differential and non-tissue-differential datasets, alternative exons that were not annotated were excluded from the analysis. Disopred3 (Jones and Cozzetto 2015) was used to predict the disordered regions within all the genes comprising these three datasets and middle exons were mapped back to these regions to determine if there was any overlap. The Shapiro-Wilk test for normality was performed on exon/intron size data and indicated that the data was not normally distributed. The statistical significance was then calculated using the Wilcoxon rank-sum test. Statistical differences in the

frequency of frame preservation, protein domain overlap and disordered region overlap were calculated using the Chi-squared test.

Additional details surrounding motif enrichment analysis

Motif analysis was performed using HOMER (Heinz et al. 2010) to discover annotated or *de novo* motifs that were enriched in tissue-regulated splicing events. One analysis involved a search for enriched motifs in all alternatively spliced cassette exons and flanking introns (370), with a background file of an equivalent region of constitutively spliced exons and flanking introns (3,151). The second analysis involved all tissue differential splicing events (775), with a background file of non-tissue- differential splicing events (1,679).

FASTA files of each pair of sequences was input into the findMotifs.pl script to find motifs of length 4, 6, 8 and 10 nucleotides. HOMER returns known RBP motifs from previous in vitro defined consensus data (Ray et al. 2013), but also many transcription factor and other DNA binding motifs. In cases of no known RBP-motif match, the enriched motifs were searched against the Hughes lab database (Ray et al. 2013; http://hugheslab.ccbr.utoronto.ca/supplementary-data/RNAcompete_eukarya/Experiment_reports/RNAcompete_report_index.html) to see if another close RBP-motif match exists.

Construction of two-color splicing reporter plasmids

We revised our previous two-color splicing reporter (Norris et al. 2014) to include several new features (Fig 2A). This new reporter (pWASR2) was synthesized (GenScript corporation) and cloned into the pUC57 vector. Several common promoters to drive expression in tissues and cells

of interest were amplified from N2 genomic DNA (pan-neuronal (*rgef-1*), body wall muscles (*myo-3*), intestine (*ges-1*), dopaminergic neurons (*dat-1*), and serotonergic neurons (*tph-1*) – see Supplemental Table S9 for a list of primers). These amplified promoters were inserted in between ClaI and KpnI restriction enzyme sites by Gibson Assembly.

To insert minigenes into promoter-containing plasmids, we amplified regions spanning the first exon, intron and 5′ end of the alternative exon by PCR using N2 genomic DNA as a template (see Supplemental Table S8 for a list of primers). gBlocks spanning the 3′ end of the alternative exon to the end of the third exon were ordered from Integrated DNA Technologies (IDT), where an additional nucleotide is added to each alternative exon and point mutations are generated to remove stop codons in the required reporter reading frames. PCR fragments and gBlocks were then inserted in between KpnI and NotI restriction enzyme sites in the appropriate promoter-containing vectors by Gibson Assembly.

To generate *zoo-1* splicing reporters with mutations in UNC-75 binding sites, using the *zoo-1* wild-type reporter as a template, we introduced point mutations via primers and PCR-amplified regions upstream and downstream of these point mutations. These PCR amplicons and then cloned in between KpnI and NotI sites of the two-color reporter plasmid. All primers were ordered from IDT, and all vectors were verified by Sanger sequencing. For a complete list of vectors used in this study, please see Supplemental Table S8.

Microinjection, integration, strains, microscopy, and quantification of two-color reporters

All plasmids were injected at concentrations of 50 ng/μL as described previously (Evans 2006) and extrachromosomal array containing animals were maintained by selecting fluorescent progeny each generation using a fluorescent stereomicroscope. For integration of some of our two-color splicing reporters, we used a recently described approach (Noma and Jin 2018) using a mini singlet oxygen generator (miniSOG) fused to a histone protein to generate double strand breaks in the genome of animals injected with our plasmids. Animals with integrated arrays were screened with a fluorescent stereomicroscope.

During the course of our analysis, we discovered that the frame-shifted 'GFP' isoform of our two-color reporters can be degraded by the NMD pathway. We believe that this NMD sensitivity arises when the stop codon at the end of the GFP ORF is utilized by the ribosome, because this creates an elongated 3′ UTR, which has previously been demonstrated in *C. elegans* to be a trigger for NMD (Longman et al. 2007). Therefore, we crossed or injected our reporters into a *smg-1* genetic background. Tissue-specific reporter splicing patterns agreed regardless of using wild-type or *smg-1* backgrounds (compare Figs 2, 7, and S2 with Fig S8). The main difference is that the GFP signal is significantly brighter in the *smg-1* background.

For microscopy, transgenic animals were mounted on slides containing 2% agarose pads and then covered with a coverslip. Microscopy was performed using a Leica SP8 laser scanning confocal microscope. In cases where reporters were expressed in multiple tissues in the same animal, we first found imaging conditions that maximized GFP and mCherry signal without saturating the image. This allowed for relative GFP/mCherry signals to be observed in each tissue within the same animal. In the case of quantification of *zoo*-1 reporter images (Fig 7F and G), we optimized

imaging conditions across the wild-type and site 1&2 mutant reporter strains to ensure GFP and mCherry signals were below saturation across all genotypes. We then imaged five L4 animals from each reporter expressing strain in the same microscopy session. After acquisition, raw images were further processed in FIJI to generate maximum intensity z-projection images, to separate channels, and to save resulting processed images. For quantification, we obtained summed pixel intensity counts for both the GFP and mCherry channels and background subtracted these counts. The resulting mCherry/GFP ratios were calculated and plotted in Fig 7G as a mean +/- one standard deviation.

Additional details for multiple sequence alignments and conservation analysis

We obtained coordinates of all relevant splicing events from our TRAP-seq data (spanning three exons and two introns where the internal exon is either an alternative or constitutive exon). Using these coordinates, corresponding BED files were generated and used to obtain multiple alignment format (MAF) files from the 26-way nematode alignment track on genome assembly ce11 (WBCel235) from the UCSC (University of California Santa Cruz) Genome Browser. We then extracted homologous sequences from seven species (*C. elegans*, *C. brenneri*, *C. briggsae*, *C. remanei*, *C. japonica*, *C. tropicalis*, and *C. angaria*) and re-aligned relevant regions surrounding alternative or constitutive exons using MUSCLE (Edgar 2004). In order to measure conservation patterns in a base-by-base manner, we used the program phyloP (Pollard et al. 2010), a part of the "rphast" R package (Hubisz et al. 2011).

Alignments were ordered such that the phyloP analysis would be *C. elegans* centric, and the phylogenetic tree and neutral model file used was obtained from the 7-way nematode alignment file available at http://compgen.cshl.edu/phast/. The distributions of phyloP values in defined

regions (upstream or downstream intronic 5′ or 3′ ends) were compared between different classes of splicing events using the Wilcoxon signed-rank test.

Supplemental Methods References

3.5.1. RDCT. 2018. A Language and Environment for Statistical Computing. *R Found Stat Comput* **2**: https://www.R-project.org. http://www.r-project.org.

Anders S, Pyl PT, Huber W. 2015. HTSeq-A Python framework to work with high-throughput sequencing data. *Bioinformatics* **31**: 166–169.

de Hoon MJL, Imoto S, Nolan J, Miyano S. 2004. Open source clustering software. *Bioinformatics* **20**: 1453–1454.

Edgar RC. 2004. MUSCLE: Multiple sequence alignment with high accuracy and high throughput. *Nucleic Acids Res* **32**: 1792–1797.

Evans T. 2006. Transformation and microinjection. *WormBook*.

Grove C, Cain S, Chen WJ, Davis P, Harris T, Howe KL, Kishore R, Lee R, Paulini M, Raciti D, et al. 2018. Using WormBase: A genome biology resource for caenorhabditis elegans and related nematodes. In *Methods in Molecular Biology*, Vol. 1757 of, pp. 399–470.

Heinz S, Benner C, Spann N, Bertolino E, Lin YC, Laslo P, Cheng JX, Murre C, Singh H, Glass CK. 2010. Simple Combinations of Lineage-Determining Transcription Factors Prime cis-Regulatory Elements Required for Macrophage and B Cell Identities. *Mol Cell* **38**: 576–589.

Hubisz MJ, Pollard KS, Siepel A. 2011. Phastand Rphast: Phylogenetic analysis with space/time models. *Brief Bioinform* **12**: 41–51.

Jones DT, Cozzetto D. 2015. DISOPRED3: Precise disordered region predictions with annotated

protein-binding activity. *Bioinformatics* **31**: 857–863.

Longman D, Plasterk RHA, Johnstone IL, Cáceres JF. 2007. Mechanistic insights and identification of two novel factors in the C. elegans NMD pathway. *Genes Dev* **21**: 1075–1085.

Noma K, Jin Y. 2018. Rapid integration of multi-copy transgenes using optogenetic mutagenesis in Caenorhabditis elegans. *G3 Genes, Genomes, Genet* **8**: 2091–2097.

Norris AD, Gao S, Norris ML, Ray D, Ramani AK, Fraser AG, Morris Q, Hughes TR, Zhen M, Calarco JA. 2014. A Pair of RNA-binding proteins controls networks of splicing events contributing to specialization of neural cell types. *Mol Cell* **54**: 946–959.

Pollard KS, Hubisz MJ, Rosenbloom KR, Siepel A. 2010. Detection of nonneutral substitution rates on mammalian phylogenies. *Genome Res* **20**: 110–121.

Ramani AK, Calarco JA, Pan Q, Mavandadi S, Wang Y, Nelson AC, Lee LJ, Morris Q, Blencowe BJ, Zhen M, et al. 2011. Genome-wide analysis of alternative splicing in Caenorhabditis elegans. *Genome Res* **21**: 342–348.

Ray D, Kazan H, Cook KB, Weirauch MT, Najafabadi HS, Li X, Gueroussov S, Albu M, Zheng H, Yang A, et al. 2013. A compendium of RNA-binding motifs for decoding gene regulation. *Nature* **499**: 172–177.

Tourasse NJ, Millet JRM, Dupuy D. 2017. Quantitative RNA-seq meta-analysis of alternative exon usage in C. elegans. *Genome Res* **27**: 2120–2128.

Figure S1

Replicate	Tissue/cell type IP	Uniquely mapped reads	Matched whole animal uniquely mapped reads
A	neurons	158,320,817	79,099,911
B	neurons	55,167,075	48,501,520
A	muscle	49,579,407	46,654,520
B	muscle	102,909,271	130,878,020
A	intestine	73,180,094	42,504,763
B	intestine	146,222,460	113,194,522
A	dopaminergic neurons	129,608,718	255,993,817
B	dopaminergic neurons	169,520,157	128,354,536
A	serotonergic neurons	103,948,246	12,764,025
B	serotonergic neurons	177,215,778	91,176,490
	**Total uniquely mapped reads**	**1,165,672,023**	**949,122,124**

Supplemental Fig S1: Summary of dataset used in the current study (Related to Figure 1)
Table displays a breakdown of each replicate sample collected, and the number of uniquely aligning reads to the C. elegans genome and/or transcriptome. The first column indicates the replicate ID, the second column represents what tissue or cell-type expressed the GFP::RPL-1 protein, the third column displays the uniquely mapped reads from cDNA libraries prepared from the TRAP immunoprecipitated RNA, and the fourth column displays the uniquely mapped reads from cDNA prepared from an aliquot of whole animal lysate prior to immunoprecipitation, matched from the same sample.

Figure S2

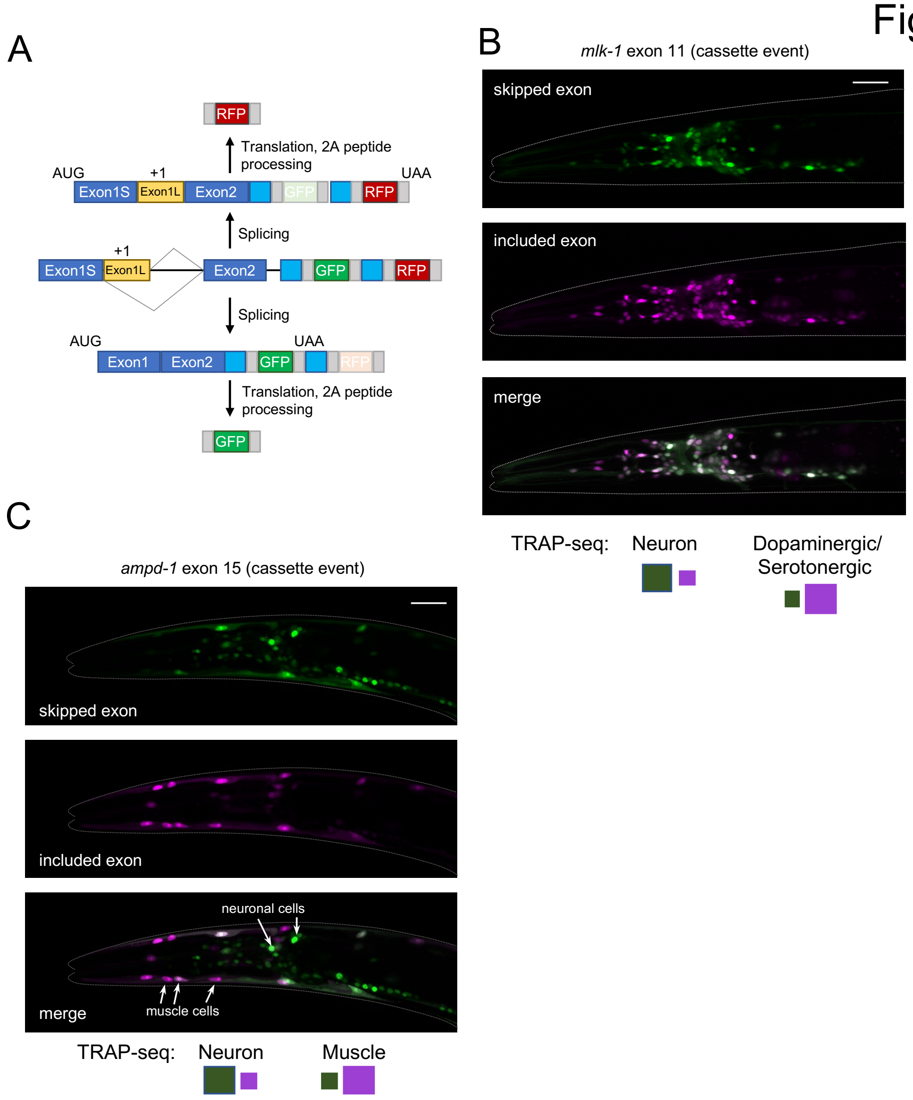

Supplemental Fig S2: Additional validation of TRAP-seq measurements with two-colour splicing reporters (Related to Figure 2)

A. Schematic of the two-colour splicing reporter architecture for alternative splice site selection. Diagram related to Figure 2A, except single nucleotide to shift reading frame is inserted in between portion of exon that is flanked by two competing splice sites (alternative 5′ splice site is shown in diagram, but alternative 3′ splice site conceptually similar).

B. Fluorescence microscopy of splicing reporter expressed in neurons monitoring a splicing event in the *mlk-1* gene. This splicing event was detected to be differentially spliced between neurons and dopaminergic/serotonergic neurons, and two colour reporters reveal differences in red/green fluorescence ratios in individual neuronal cells. Top labels: Gene, alternatively spliced exon and splicing class.

C. Same as in A, with splicing reporter expressed in neurons and muscle cells monitoring splicing event in *ampd-1* gene, measured to have increased exon skipping in neurons (more green signal), and more inclusion in muscle cells (more purple signal).

Figure S3

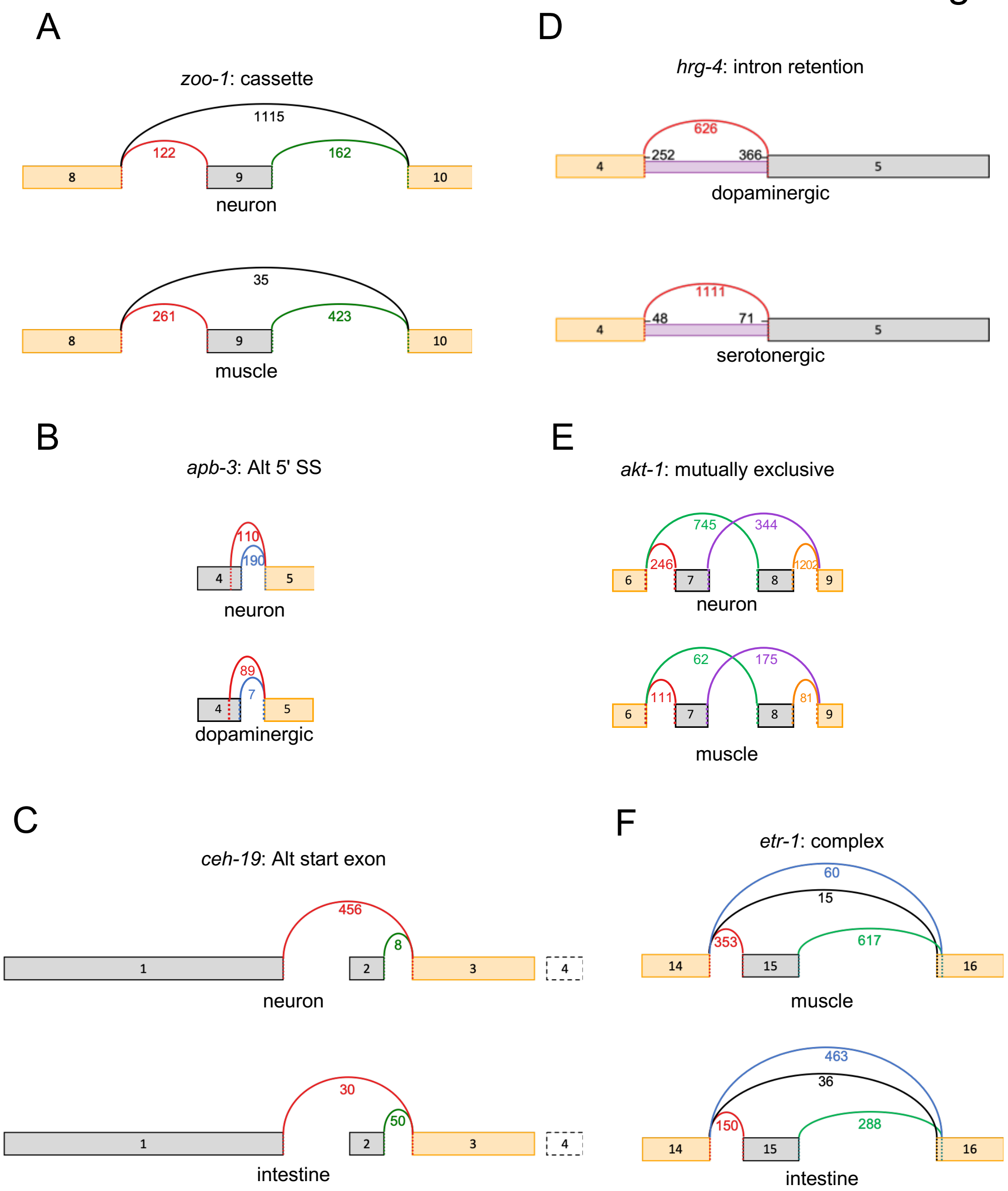

Supplemental Fig S3: Examples of detected tissue-regulated splicing events (Related to Figure 3) A-F highlight different classes of alternative junction usage identified, presented in the Voila splice graph format. boxes represent exons (yellow boxes are constitutive portions of exons, and grey boxes are alternative). Curved lines represent splice junctions connecting the exons, and numbers are read counts supporting the corresponding junctions. Examples including A) a cassette type exon skipping event in *zoo-1* B) An alternative 5′ splice site selection event in *apb-3* C) an alternative first exon event in *ceh-19* D) an intron retention event in *hrg-4* E) a mutally exclusive splicing event in *akt-1* F) a complex splicing event in *etr-1*.

Figure S4

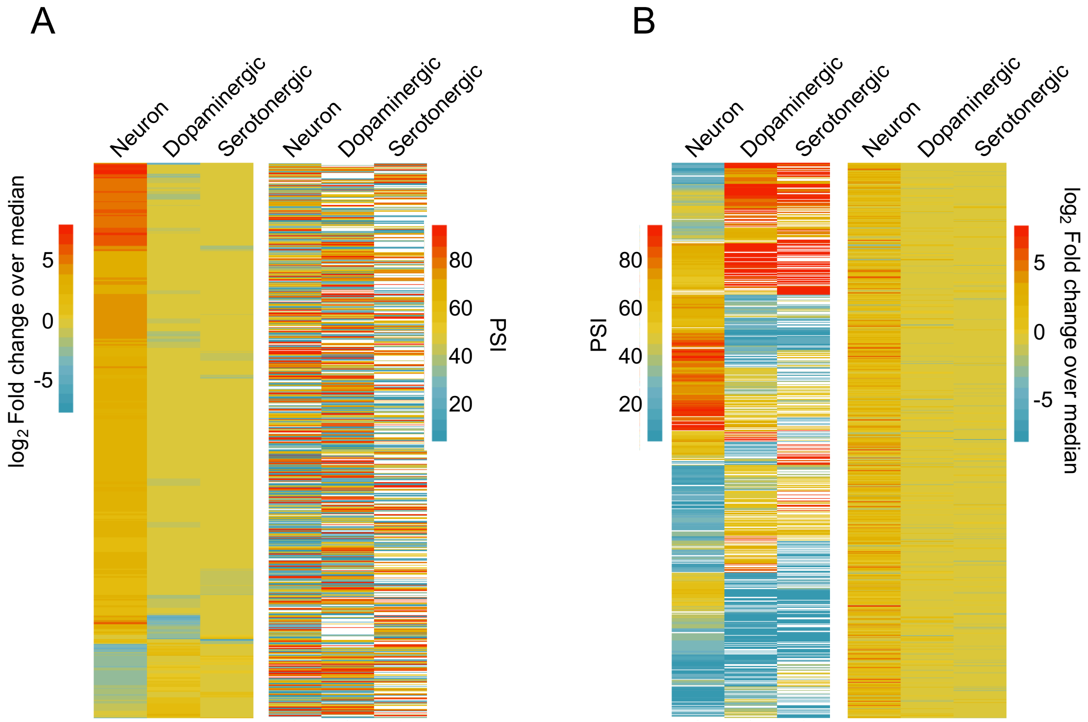

Supplemental Fig S4: Neuron-subtype-regulated splicing and steady state level differences occur in largely non-overlapping gene sets (Related to Figure 4)

A. Left panel: Genes with significant steady state transcript level differences clustered by their log2 transformed fold change over median ratios. Right panel: PSI value measurments for alternative splicing events in the same genes ordered according to panel on the left.

B. Left panel: Genes with tissue-regulated splicing patterns clustered by their respective PSI value measurements. Right panel: log2 transformed fold change over median ratios for the same genes with splicing differences, ordered according to panel on the left.

Figure S5

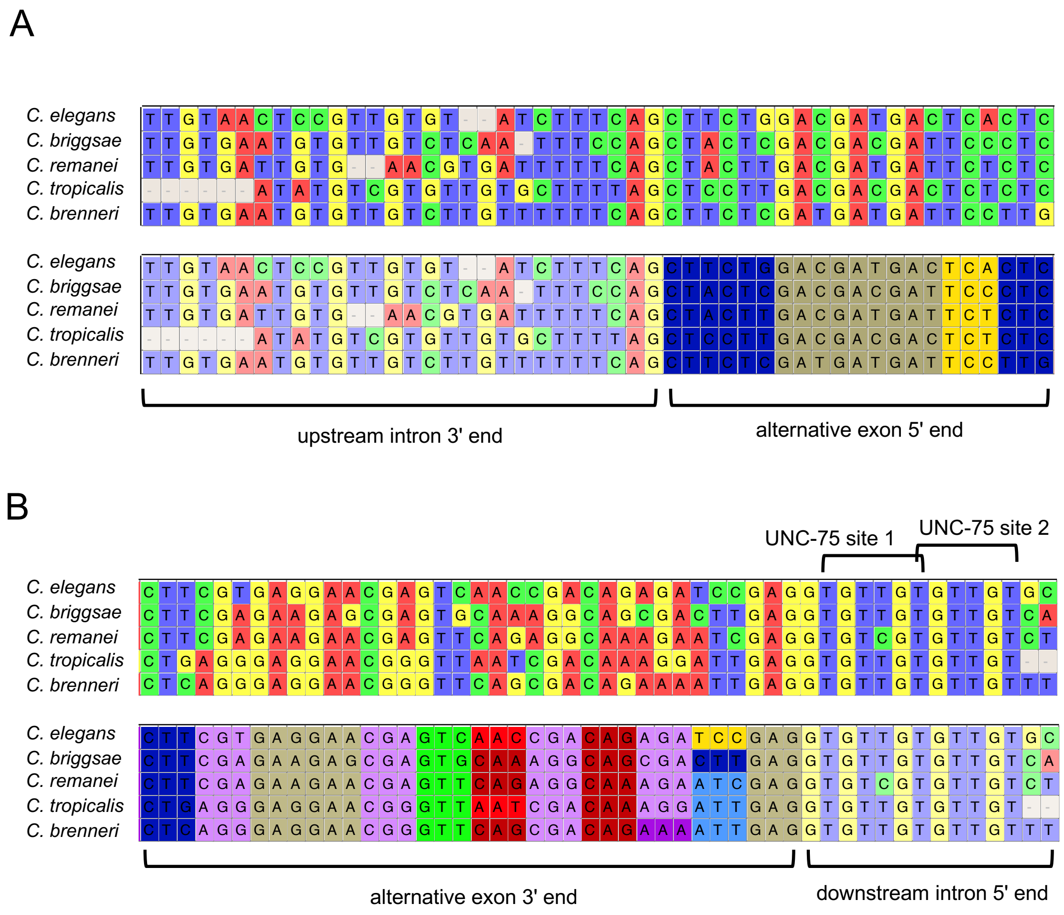

Supplemental Fig S5: Conservation of UNC-75/CELF consensus *cis*-elements overlapping a 5′ splice site (Related to Figure 7)

A. Multiple sequence alignment of *zoo-1* 5′ portion of alternative exon 9 and 3′ portion of the upstream flanking intron. Top panel is colour-coded by nucleotide. Bottom panel is the same sequence, but the coding exon is colour-coded by corresponding amino acid.

B. Multiple sequence alignment of *zoo-1* 3′ portion of alternative exon 9 and 5′ portion of the downstream flanking intron. Colour-coding is the same as described for panel A. Note the additional labeling of two consensus UNC- 75 binding motifs overlapping 5′ splice site.

Figure S6

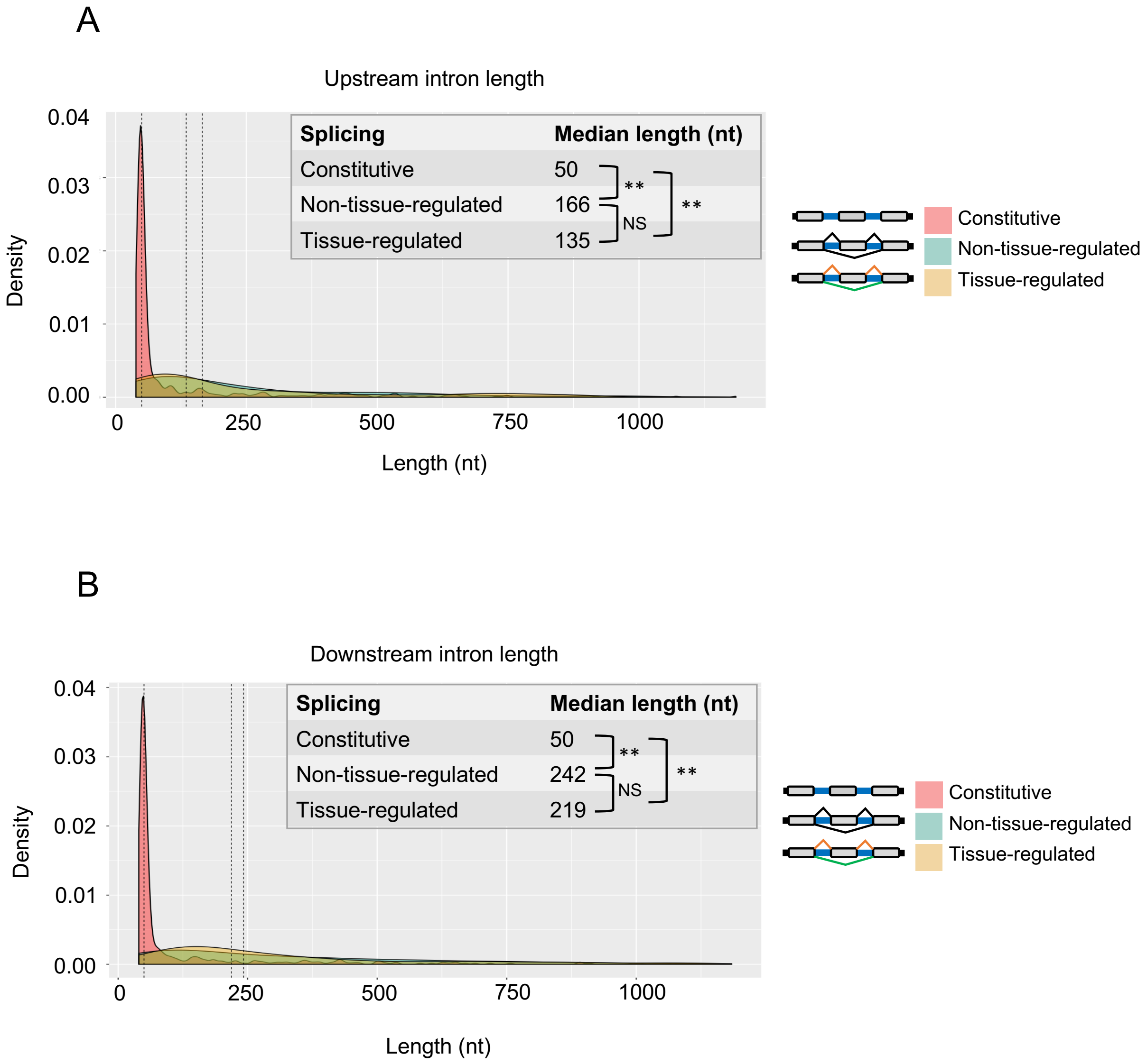

Supplemental Fig S6: Alternative exons and tissue-regulated alternative exons are flanked by longer introns compared with constitutive exons (Related to Figure 8)

For panels A and B, comparisons were made between constitutively spliced exons (pink), alternative exons (light blue), and tissue-regulated alternative exons (gold).

A. Distributions of the lengths of introns flanking internal exons from same comparison groups

B. Distribution of the lengths of exons

For A-B: Wilcoxon rank-sum test. NS = not significant, **p < 1 x 10-15

Figure S7

A

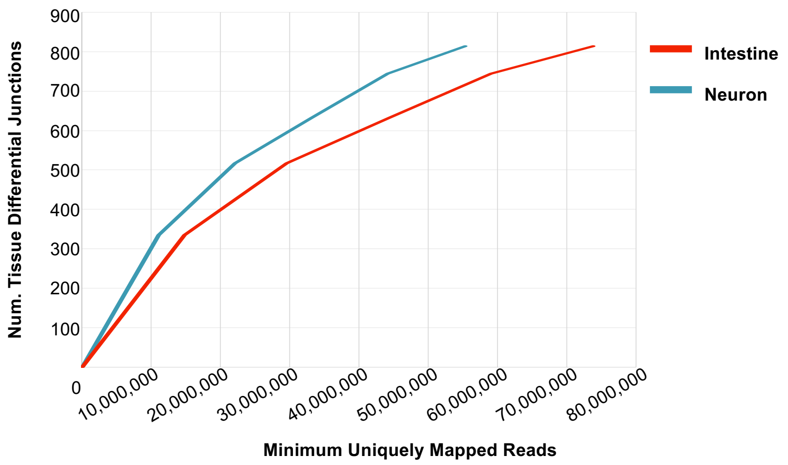

B

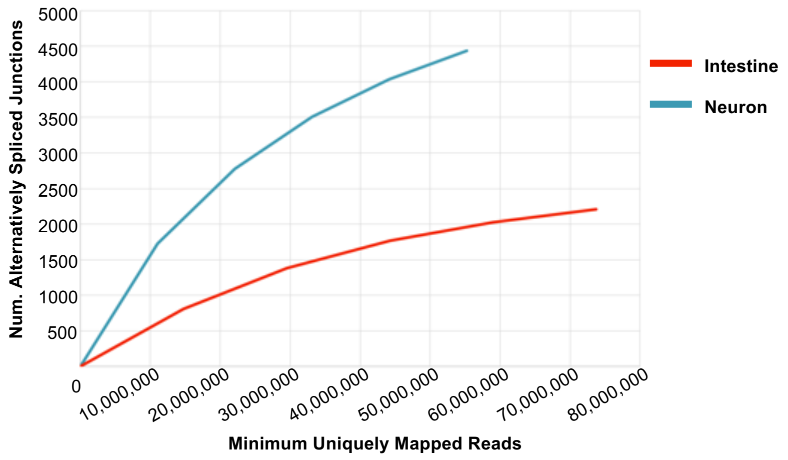

Supplemental Fig S7: Down-sampling of initial sequencing data to assess relationship between depth of sequencing and detection of alternatively spliced junctions (Related to Figures 1 and 3)

A. Number of splice junctions detected as differentially-regulated between neuronal and intestinal TRAP-seq data as a function of the minimum number of uniquely mapped reads in each replicate from that tissue.

B. Number of splice junctions detected as alternatively spliced from our neuronal and intestinal TRAP-seq data as a function of the minimum number of uniquely mapped reads in each replicate from that tissue.

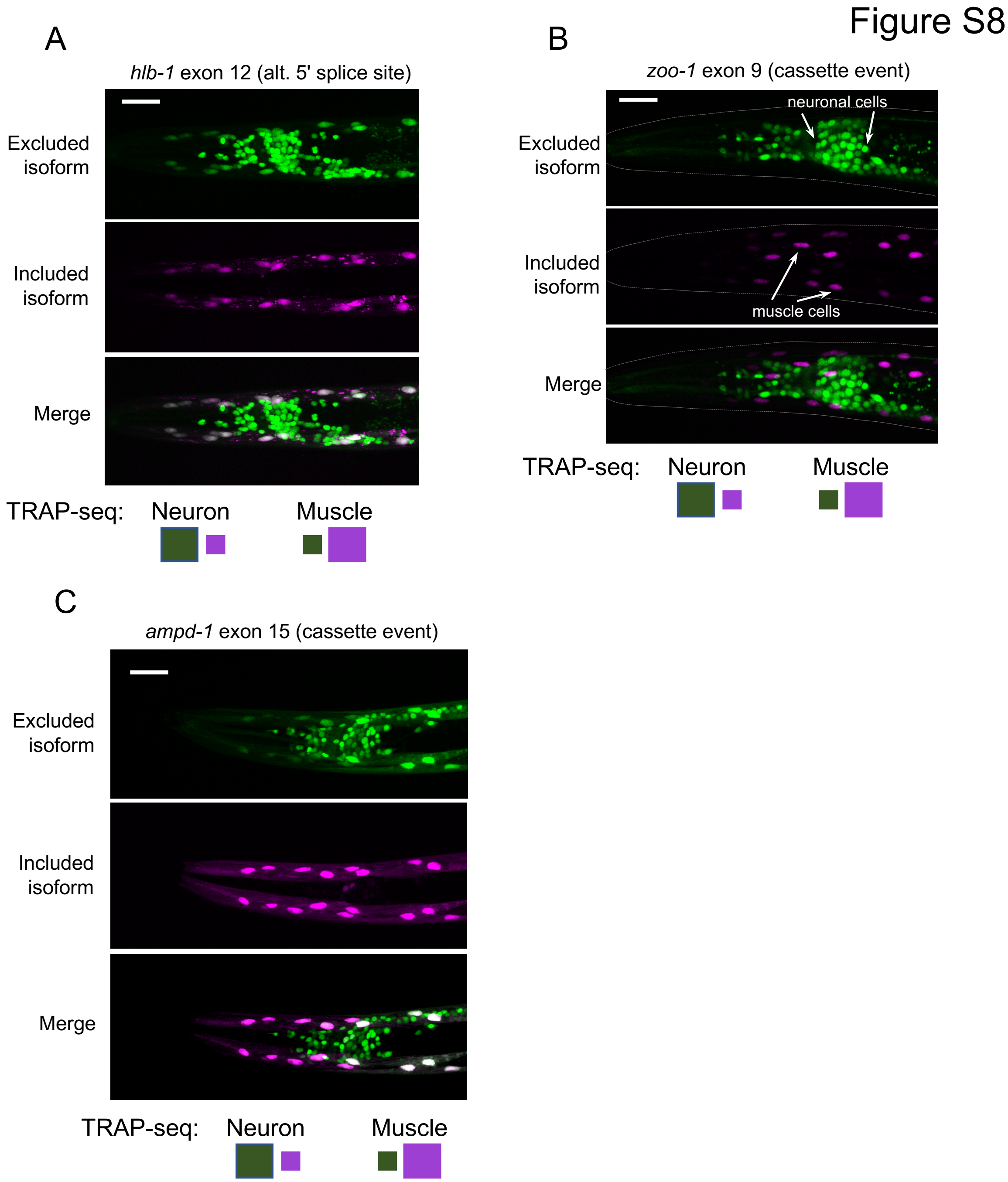

Supplemental Fig S8: Two-colour splicing reporters imaged in a wild-type genetic background (Related to Figure 2 and Figure S2)

A-C show examples of two-colour reporters expressed in neuronal and body wall muscle cells and imaged in wild-type animals as opposed to a *smg-1* genetic background. Tissue-specific splicing patterns are recapitulated, however, the GFP signal is weaker due to reduced stability and thus requires additional laser power and gain settings.
